# Subcutaneous BCG vaccination protects against streptococcal pneumonia via regulating innate immune responses in the lung

**DOI:** 10.15252/emmm.202217084

**Published:** 2023-05-09

**Authors:** Alisha Kang, Gluke Ye, Ramandeep Singh, Sam Afkhami, Jegarubee Bavananthasivam, Xiangqian Luo, Maryam Vaseghi‐Shanjani, Fatemah Aleithan, Anna Zganiacz, Mangalakumari Jeyanathan, Zhou Xing

**Affiliations:** ^1^ McMaster Immunology Research Centre, M. G. DeGroote Institute for Infectious Disease Research McMaster University Hamilton ON Canada; ^2^ Department of Medicine McMaster University Hamilton ON Canada; ^3^ Department of Pediatric Otolaryngology, Shenzhen Hospital Southern Medical University Shenzhen China

**Keywords:** lung, *Streptococcus pneumoniae* infection, subcutaneous BCG vaccine, tissue‐resident macrophages, trained innate immunity, Immunology, Microbiology, Virology & Host Pathogen Interaction, Respiratory System

## Abstract

Bacillus Calmette‐Guérin (BCG) still remains the only licensed vaccine for TB and has been shown to provide nonspecific protection against unrelated pathogens. This has been attributed to the ability of BCG to modulate the innate immune system, known as trained innate immunity (TII). Trained innate immunity is associated with innate immune cells being in a hyperresponsive state leading to enhanced host defense against heterologous infections. Both epidemiological evidence and prospective studies demonstrate cutaneous BCG vaccine‐induced TII provides enhanced innate protection against heterologous pathogens. Regardless of the extensive progress made thus far, the effect of cutaneous BCG vaccination against heterologous respiratory bacterial infections and the underlying mechanisms still remain unknown. Here, we show that s.c. BCG vaccine‐induced TII provides enhanced heterologous innate protection against pulmonary *Streptococcus pneumoniae* infection. We further demonstrate that this enhanced innate protection is mediated by enhanced neutrophilia in the lung and is independent of centrally trained circulating monocytes. New insight from this study will help design novel effective vaccination strategies against unrelated respiratory bacterial pathogens.

## Graphical abstract

Subcutaneous BCG vaccine‐induced trained innate immunity in the lung provides enhanced heterologous innate protection against pulmonary *Streptococcus pneumoniae* infection. This enhanced protection is mediated by increased neutrophilia in the lung and is independent of trained circulating monocytes.

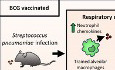

**The paper explained**

1 **Problem**

Over the years, epidemiological evidence has suggested that Bacillus Calmette‐Guérin (BCG), the only licensed vaccine for TB, can provide nonspecific protection against unrelated pathogens. This heterologous protection has been attributed to trained innate immunity (TII). BCG vaccination has been shown to protect against various infections and is linked to its activating effects on circulating monocytes, neutrophils, and NK cells via epigenetic and metabolic modifications leading to increased production of pro‐inflammatory cytokines. BCG‐induced persisting changes is associated with its training effects on hematopoietic cells in the bone marrow. In addition to centrally induced training in monocytes, cutaneous BCG vaccination can also train lung tissue‐resident macrophages via the gut–lung axis. However, regardless of the progress in the field, there is a paucity in preclinical studies of cutaneous BCG vaccination and lung TII, which has hindered the mechanistic understanding of cutaneous BCG vaccine‐induced TII in humans.

**Results**

Our study aimed to address whether subcutaneous (s.c.) BCG vaccination provides heterologous protection against respiratory bacterial infection and to investigate the underlying mechanisms. Subcutaneous BCG vaccination provided enhanced protection against pulmonary *Streptococcus pneumoniae* (*S. pneumoniae*) infection. Airway‐resident alveolar macrophages of s.c. BCG‐vaccinated hosts exhibited heightened bactericidal activity and improved protection against *S. pneumoniae* infection. This enhanced heterologous innate protection was mediated by augmented neutrophilia in the lung of s.c. BCG‐vaccinated hosts, independent of centrally trained circulating monocytes. In s.c. BCG‐vaccinated hosts, the contribution of circulating monocytes was compensated for by enhanced responses from both alveolar macrophages and neutrophils.

**Impact**

Overall, this study demonstrates that cutaneous BCG vaccination can provide protection against heterologous pulmonary *S. pneumoniae* infection via TII and increased neutrophilia in the lung and is independent of centrally trained monocytes. Such knowledge shall help design novel effective vaccination strategies against heterologous respiratory pathogens.

## Introduction

Bacillus Calmette‐Guérin (BCG), the only licensed vaccine for tuberculosis (TB), remains the mostly widely administered vaccine worldwide given via the skin (Moliva *et al*, 2017). Although it has limited efficacy in protection against pulmonary TB in adults, BCG vaccination has been found to provide nonspecific protective effects against unrelated heterologous infections beyond TB in humans (Netea *et al*, 2016; Pasco & Anguita, 2020; Xing *et al*, 2020). This has been attributed to the ability of live attenuated BCG to modulate the innate arm of the immune system and has been termed trained innate immunity (TII) (Netea *et al*, 2016). Trained innate immunity is associated with a state of hyperresponsiveness and leads to enhanced host defense (Xing *et al*, 2020).

The identification of beneficial nonspecific protective effects of BCG came from epidemiological evidence in which cutaneous BCG vaccination reduced all‐cause mortality in low‐birth‐weight infants and infants with a BCG scar had significantly lower mortality in early childhood (Garly *et al*, 2003; Jensen *et al*, 2015). In part, it is due to the reduced risk of developing lower respiratory tract infections unrelated to TB (Stensballe *et al*, 2005; De Castro *et al*, 2015). Indeed, in prospective clinical studies, cutaneous BCG vaccination was found to protect healthy adults from yellow fever virus and malaria and reduced the incidence of respiratory infections in elderly people (Arts *et al*, 2018; Walk *et al*, 2019; Giamarellos‐Bourboulis *et al*, 2020; Faustman *et al*, 2022). BCG vaccine‐mediated nonspecific innate immune protection is linked to its activating effects on circulating innate immune cells in the peripheral blood including monocytes, neutrophils, and NK cells associated with epigenetic and metabolic modifications and increased production of pro‐inflammatory cytokines (Kleinnijenhuis *et al*, 2012, 2014; Moorlag *et al*, 2020; Soto *et al*, 2022). Since most of the circulating innate immune cells such as monocytes are mature and thus have a short half‐life, BCG vaccine‐induced persisting changes in circulating innate immune cells has been found to result from its training effects on hematopoietic progenitor cells in the bone marrow (Kaufmann *et al*, 2018; Cirovic *et al*, 2020).

Preclinical studies have also demonstrated TII by parenteral or respiratory mucosal routes of BCG vaccination against heterologous pathogens including *Candida albicans*, *Streptococcus pneumoniae* and influenza virus or the target pathogen *Mycobacterium tuberculosis* (Kleinnijenhuis *et al*, 2012; Kaufmann *et al*, 2018; Mata *et al*, 2021; Vierboom *et al*, 2021; Jeyanathan *et al*, 2022). Of note, however, most of the preclinical parenteral BCG studies have demonstrated the induction of TII only following intravenous (i.v.) inoculation of BCG vaccine (Kaufmann *et al*, 2018, 2022). While few studies have made a head‐to‐head comparison with cutaneous BCG vaccination, they conclude that different from i.v. or respiratory mucosal routes of vaccination, cutaneous BCG vaccination is ineffective in TII induction (Kaufmann *et al*, 2018; Mata *et al*, 2021; Vierboom *et al*, 2021). Contrary to these observations by others, we have recently reported that in addition to centrally induced training in circulating monocytes, cutaneous BCG vaccination can also induce innate immune memory in lung‐resident macrophages via the gut–lung axis involving alterations in the intestinal microbiota‐derived metabolites (Jeyanathan *et al*, 2022). Such discrepancies between other studies and ours appear to be due to variations in the animal models including BCG vaccine dose and the timepoint postvaccination chosen to study. For instance, the BCG dose used in many of these studies is much higher (10^6^ CFU) compared with the dose used in our study (4–5 × 10^4^ CFU). We have also shown that cutaneous BCG‐induced TII is time‐dependent as at least until 2‐weeks postvaccination no alterations in lung resident macrophages were evident. Thus, up to date, most of the preclinical observations have been at odds with human studies in that clinically or in real‐world practice, BCG vaccine is always administered via the skin. It still remains unclear whether cutaneous BCG vaccination‐induced TII in the lung could enhance innate protection against heterologous respiratory bacterial pathogens and if so, what would be the underlying mechanisms.

To fill the above knowledge gap, in this study, we have used a murine model to study subcutaneous (s.c.) BCG vaccine‐induced TII in the lung. We show that s.c. BCG vaccination with a small‐dose inoculum induces TII in the lung which translates to enhanced innate immune protection against pulmonary heterologous *Streptococcus pneumoniae* (*S. pneumoniae*) infection. We provide evidence that s.c. BCG vaccine‐induced anti‐streptococcal TII is mediated through increased neutrophilia in the lung. We further demonstrate that such enhanced protection is independent of centrally trained circulating monocytes. Our study sheds new light on the mechanisms via which cutaneous BCG vaccination offers TII against heterologous respiratory pathogens in mouse lungs.

## Results

### Enhanced innate immune protection against pulmonary *S. pneumoniae* infection in BCG‐vaccinated hosts

**Figure 1:**
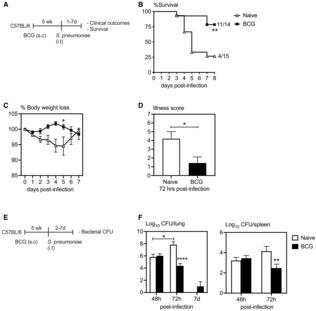
Enhanced protection against pulmonary *Streptococcus pneumoniae* infection in BCG‐vaccinated hosts Experimental schema. 5 week s.c. BCG‐vaccinated (BCG) or unvaccinated (Naïve) mice were infected with *S. pneumoniae* intratracheally and monitored for clinical outcomes.Kaplan–Meier curve showing % of mice surviving *S. pneumoniae* infection. Data was analyzed using Kaplan–Meier statistical analysis. *N =* 5–10 mice/group.Line graphs comparing changes in body weight following *S. pneumoniae* infection in BCG‐vaccinated and unvaccinated hosts. *N =* 5–10 mice/group.Bar graphs comparing clinical symptoms as a measure of illness score at 72 h post‐*S. pneumoniae* infection. *N =* 5–10 mice/group.Experimental schema, 5 week s.c. BCG‐vaccinated or unvaccinated mice were infected with *S. pneumoniae* intratracheally and bacterial burden was assessed.Bar graphs comparing bacterial burden (CFU) in the lung and spleen at 48 h, 72 h and 7 days post‐*S. pneumoniae* infection in unvaccinated and BCG‐vaccinated mice. *N =* 3–5 mice/group. Data information: Data presented in (B–D and F) show mean ± SEM of biological replicates. Statistical analysis for (C and F) were two‐way ANOVA with Sidak's multiple comparisons test and data in (D) was analyzed by two‐tailed unpaired *t*‐test. *P < 0.05; **P < 0.01; ****P < 0.0001. Source data are available online for this figure.

To begin addressing subcutaneous (s.c.) BCG vaccine‐induced TII in the lung, we used a murine model of heterologous *S. pneumoniae* respiratory infection. C57BL/6 mice vaccinated s.c. with BCG for 5 weeks were infected intratracheally (i.t.) with *S. pneumoniae* serotype 3 and evaluated for survival and clinical outcomes as a measure of weight loss and clinical signs (Fig 1A). A set of unvaccinated mice were also infected with *S. pneumoniae* as controls. Compared with control mice, a significantly greater number of s.c. BCG‐vaccinated mice survived the infection (Fig 1B). While ~ 75% of BCG‐vaccinated mice survived the infection, only ~ 25% of control mice survived the infection. Of interest, BCG‐vaccinated mice hardly lost any weight during the infection and exhibited minimal clinical signs. In stark contrast, a significant number of unvaccinated mice reached end point by 5‐days postinfection (~ 70%) and exhibited severe clinical signs (Figs 1B–D). We next assessed the bacterial burden in the lung and spleen by colony‐forming units (CFU) assay (Fig 1E). Keeping in line with significantly improved clinical outcomes, s.c. BCG‐vaccinated mice better controlled bacterial replication both locally in the lung and systemically in the spleen as indicated by reduced bacterial burden at 72‐h postinfection (Fig 1F). BCG‐vaccinated mice carried ~ 1.5 log less bacteria in the lung and spleen compared with their control counterparts. Of note, while lung bacterial counts continued to increase in control mice (significantly increased CFU at 72‐h compared to 48‐h), bacterial burden was comparable or slightly lower at 72‐h compared to 48‐h in BCG‐vaccinated hosts. To evaluate whether bacterial infection in the lung of the vast majority of surviving BCG‐vaccinated animals would continue to dwindle, 7‐days postinfection bacterial burden in the lung of s.c. BCG‐vaccinated mice was determined. Compared to 72‐h postinfection, only a very small number of bacteria were detected from the lung at 7‐days postinfection (Fig 1F), indicating effective clearance of infection in BCG‐vaccinated mice. If this trend were to continue, complete clearance would be expected in the subsequent days. Next, we evaluated the longevity of BCG‐mediated protection against pulmonary *S. pneumoniae* infection using C57BL/6 mice vaccinated with s.c. BCG for 16 weeks (Appendix Fig S1A). Although both groups lost weight to a similar extent, illness related symptoms tend to be lower in BCG‐vaccinated hosts (Appendix Fig S1B and C). Keeping in line with reduced illness related symptoms, bacterial burden in the lung and spleen of BCG‐vaccinated hosts was ~ 1 log less compared with naïve counterparts (Appendix Fig S1D). The above data indicate that subcutaneous BCG vaccination leads to effective heterologous protection against pulmonary *S. pneumoniae* infection in the lung.

### Enhanced neutrophilia in the airways following pulmonary *S. pneumoniae* infection in BCG‐vaccinated hosts

**Figure 2:**
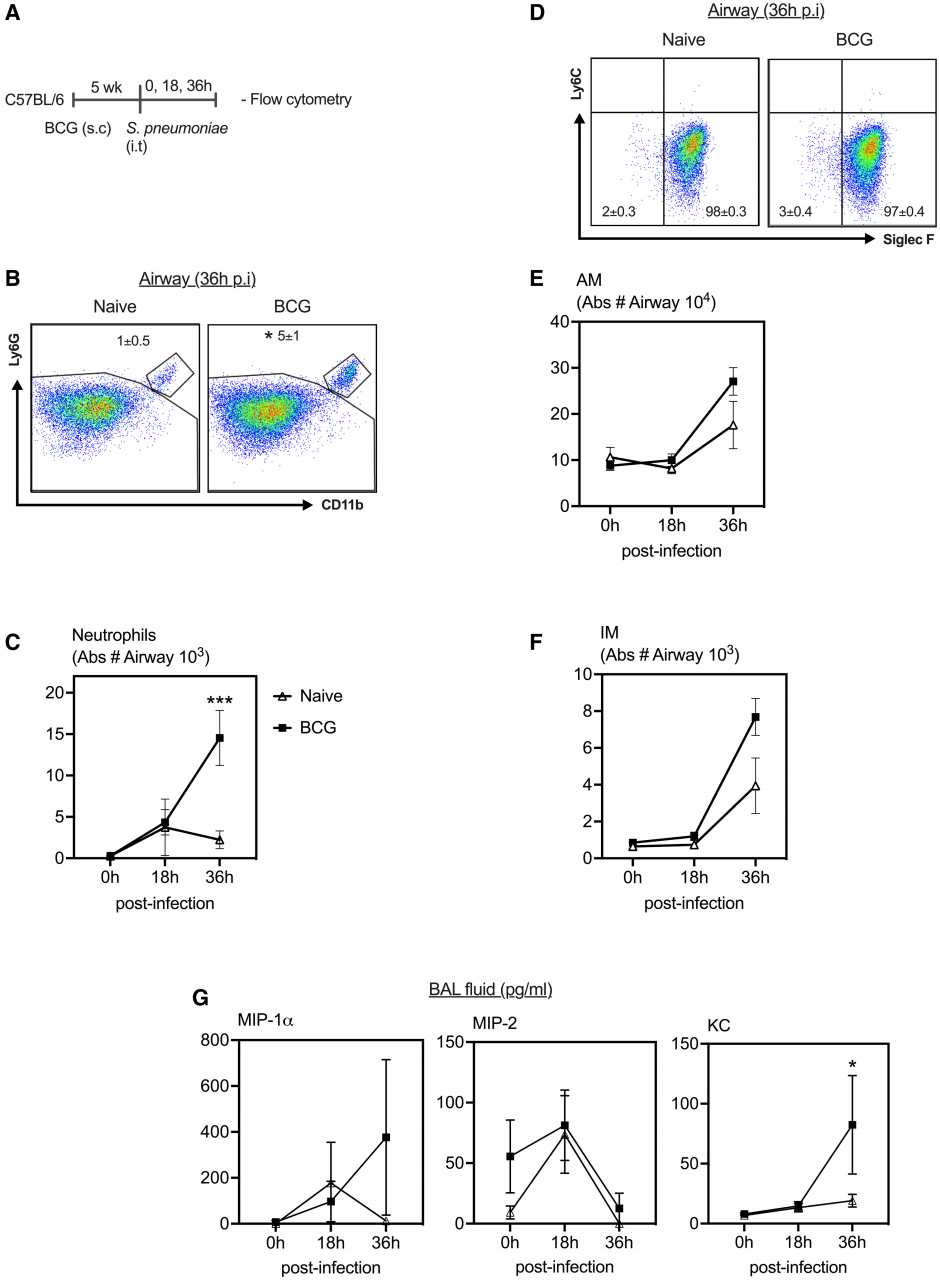
Enhanced neutrophilia in the airways following pulmonary *Streptococcus pneumoniae* infection in BCG‐vaccinated hosts A
Experimental schema. 5 week s.c. BCG‐vaccinated or naive mice were infected with *S. pneumoniae* i.t. and flow cytometry was carried out in the airways and lung.B
Dot plots of gated mononuclear cells from the BAL of control and BCG‐vaccinated mice. Analysis was performed utilizing default FlowJo V.10 software settings.C
Line graphs comparing absolute neutrophil cell counts in the airways at 18 and 36 h post‐*S. pneumoniae* infection in BCG‐vaccinated and unvaccinated hosts. *N =* 3–4 mice/group.D
Dot plots of gated mononuclear cells from the airways of control and BCG‐vaccinated hosts. Analysis was performed utilizing default FlowJo V.10 software settings.E, F
Line graphs comparing absolute AM and IM cell counts in the airways at 18 and 36 h post‐*S. pneumoniae* infection in BCG‐vaccinated and unvaccinated hosts. *N =* 3–4 mice/group.G
Line graphs comparing the levels of neutrophilic chemokines in the BAL fluid at 18 and 36 h post‐*S. pneumoniae* infection in BCG‐vaccinated and unvaccinated hosts. *N =* 3–4 mice/group. Data information: Data presented in (B–G) represent mean ± SEM of biological replicates. Statistical analysis for (C, E–G) were two‐way ANOVA with Sidak's multiple comparisons test. *P < 0.05; ***P < 0.001. Source data are available online for this figure.

We next evaluated the potential mechanisms underlying enhanced protection in BCG‐vaccinated hosts. We first assessed cellular immune responses in the airways at early time points following pulmonary *S. pneumoniae* infection. To this end, cellular responses were profiled before (0‐h) and at 18‐ and 36‐h post‐*S. pneumoniae* infection in the airways and lung of BCG‐vaccinated and control hosts (Fig 2A) using a well‐established gating strategy (Appendix Fig S2). Airway cells isolated by bronchoalveolar lavage (BAL) and lung tissue mononuclear cells were immunostained using a comprehensive panel of innate immune surface markers as described previously and analyzed by flow cytometry (Yao *et al*, 2018). The airways were predominately comprised of alveolar macrophages (AM) both in control and in BCG‐vaccinated hosts before *S. pneumoniae* infection (0‐h) (data not shown). Upon infection, while the total number of neutrophils were comparably increased in the two groups at 18‐h, they were markedly greater in the airways of BCG‐vaccinated hosts at 36‐h postinfection than in control animals (Fig 2B and C). In comparison, the total number of AM and interstitial macrophages (IM) increased postinfection in the airways of both groups with only marginally increased numbers in BCG‐vaccinated hosts at 36‐h postinfection (Fig 2D–F).

**Figure 3:**
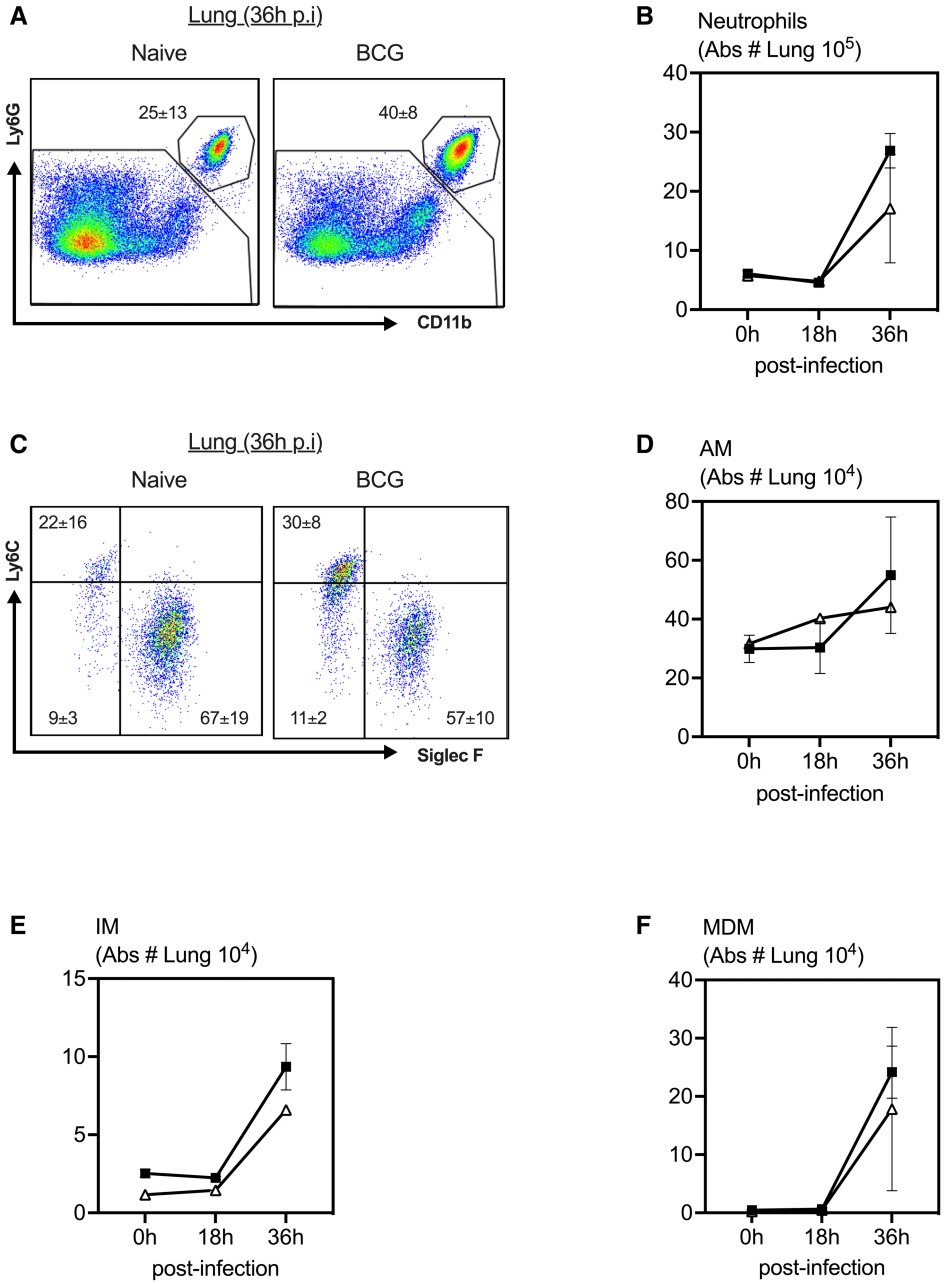
Comparable cellular responses in the lung following *Streptococcus pneumoniae* infection in control and BCG‐vaccinated hosts A
Dot plots of gated mononuclear cells from the lung of control and BCG‐vaccinated hosts. Analysis was performed utilizing default FlowJo V.10 software settings.B
Line graphs comparing absolute neutrophil cell counts in the lung at 18 and 36 h post‐*S. pneumoniae* infection in BCG‐vaccinated and unvaccinated hosts. *N =* 3–4 mice/group.C
Dot plots of gated mononuclear cells from the lung of control and BCG‐vaccinated hosts. Analysis was performed utilizing default FlowJo V.10 software settings.D–F
Line graphs comparing absolute AM, IM and MDM cell counts in the lung at 18 and 36 h post‐*S. pneumoniae* infection in BCG‐vaccinated and unvaccinated hosts. *N =* 3–4 mice/group. Data information: Data presented in (A–F) represent mean ± SEM of biological replicates. Statistical analysis for (B, D–F) were two‐way ANOVA with Sidak's multiple comparisons test. Source data are available online for this figure.

Given much enhanced neutrophilia in the airways of BCG‐vaccinated hosts, we examined neutrophil attracting chemokine protein levels in the BAL fluids at various time points postinfection. As expected, before infection (0‐h), the levels of KC, MIP‐2, and MIP‐1α were minimal in the airways of both control and BCG‐vaccinated hosts (Fig 2G). Following infection, these chemokine levels were largely comparable between the two groups at 18‐h postinfection (Fig 2G). However, in keeping with increased neutrophil recruitment to the airways at 36‐h postinfection (Fig 2C), neutrophil attracting chemokine, KC protein levels were significantly higher in the airways of BCG‐vaccinated hosts compared with the controls at 36‐h postinfection (Fig 2G). On the contrary, cellular responses including neutrophils, AM, IM, and monocyte‐derived macrophages (MDM) in the lung tissue did not differ between groups and they increased in both groups at 36‐h postinfection (Fig 3A–F). The above data suggest an association between enhanced airway neutrophilia and improved protection against pulmonary *S. pneumoniae* infection in subcutaneous BCG‐vaccinated hosts.

### Heightened bactericidal activity and improved protection against *S. pneumoniae* infection mediated by AMs from BCG‐vaccinated hosts

**Figure 4:**
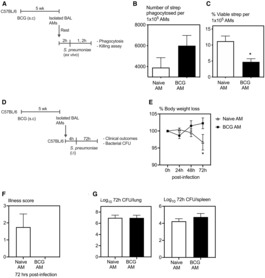
Heightened bactericidal activity and improved protection against *Streptococcus pneumoniae* infection mediated by AMs from BCG‐vaccinated hosts Experimental schema. Alveolar macrophages were isolated from 5 week s.c. BCG‐vaccinated or naive mice followed by *ex vivo S. pneumoniae* infection and their functionality was assessed.Bar graphs comparing phagocytosis as intracellular CFU of *S. pneumoniae* by AMs isolated from unvaccinated and BCG‐vaccinated hosts. *N =* 3 replicates/group.Bar graphs comparing the killing rates of phagocytosed *S. pneumoniae* by AMs at 1 and 2 h post‐*ex vivo S. pneumoniae* infection. *N =* 3 replicates/group.Experimental schema. Alveolar macrophages were isolated from 5 week s.c. BCG‐vaccinated or naive donor mice, adoptively transferred to recipient naïve mice followed by i.t. *S. pneumoniae* infection. Mice were monitored for clinical outcomes and bacterial burden was assessed.Line graphs comparing changes in body weight following *S. pneumoniae* infection in BCG‐vaccinated and unvaccinated hosts. *N =* 4 mice/group.Bar graphs comparing clinical symptoms as a measure of illness score at 72 h post‐*S. pneumoniae* infection. *N =* 4 mice/group.Bar graphs comparing bacterial counts (CFU) in the lung and spleen at 72 h post‐*S. pneumoniae* infection in BCG‐vaccinated and unvaccinated hosts. *N =* 4–5 mice/group. Data information: Data presented in (B, C, E–G) represent mean ± SEM of technical replicates. Statistical analysis for (B, C, F and G) were two‐tailed unpaired *t*‐tests, for (E) was two‐way ANOVA with Sidak's multiple comparisons test. *P < 0.05. Source data are available online for this figure.

Since alveolar macrophages are part of the first line of defense against respiratory tract infections and have been shown to play a vital role in protection against *S. pneumoniae* infection (Knapp *et al*, 2003; Yao *et al*, 2018; Kulikauskaite & Wack, 2020), we next evaluated the protective role of AMs from BCG‐vaccinated hosts first by using an *ex vivo* approach. To this end, AMs harvested by bronchoalveolar lavage from the airways of control (Naïve‐AM) and 5‐week BCG‐vaccinated mice (BCG‐AM) were infected *ex vivo* with *S. pneumoniae* (Fig 4A). Phagocytic capacity and killing rates were determined at 1‐ and 2‐h post‐*ex vivo* infection, respectively, by CFU assay. Indeed, compared with Naive‐AMs, BCG‐AMs appeared to have phagocytosed somewhat more bacteria (Fig 4B) and demonstrated significantly increased intracellular bacterial killing rates (Fig 4C).

We next used an AM adoptive transfer approach to assess the role of AMs in enhanced protection against *S. pneumoniae* infection *in vivo*. Thus, following our previously documented protocol (Yao *et al*, 2018), the AMs harvested from the airways of control and BCG‐vaccinated donor mice were adoptively transferred to naïve mice. At 4‐h after transferring AMs, these mice were infected with *S. pneumoniae* and clinical outcomes and bacterial burden were assessed (Fig 4D). BCG‐AM transferred mice demonstrated significantly improved protection compared with those transferred with Naïve‐AM by sustaining their body weight and without showing any clinical signs of disease (Fig 4E and F). Of interest, bacterial burden in the lung and spleen were comparable between the two groups at 72‐h postinfection (Fig 4G). The above data together suggest that airway‐resident alveolar macrophages of subcutaneous BCG‐vaccinated hosts contribute to trained innate immunity against heterologous *S. pneumoniae* infection in the lung.

### Enhanced neutrophilia during the early stages of infection plays a protective role against *S. pneumoniae* infection in the lung of BCG‐vaccinated hosts

**Figure 5:**
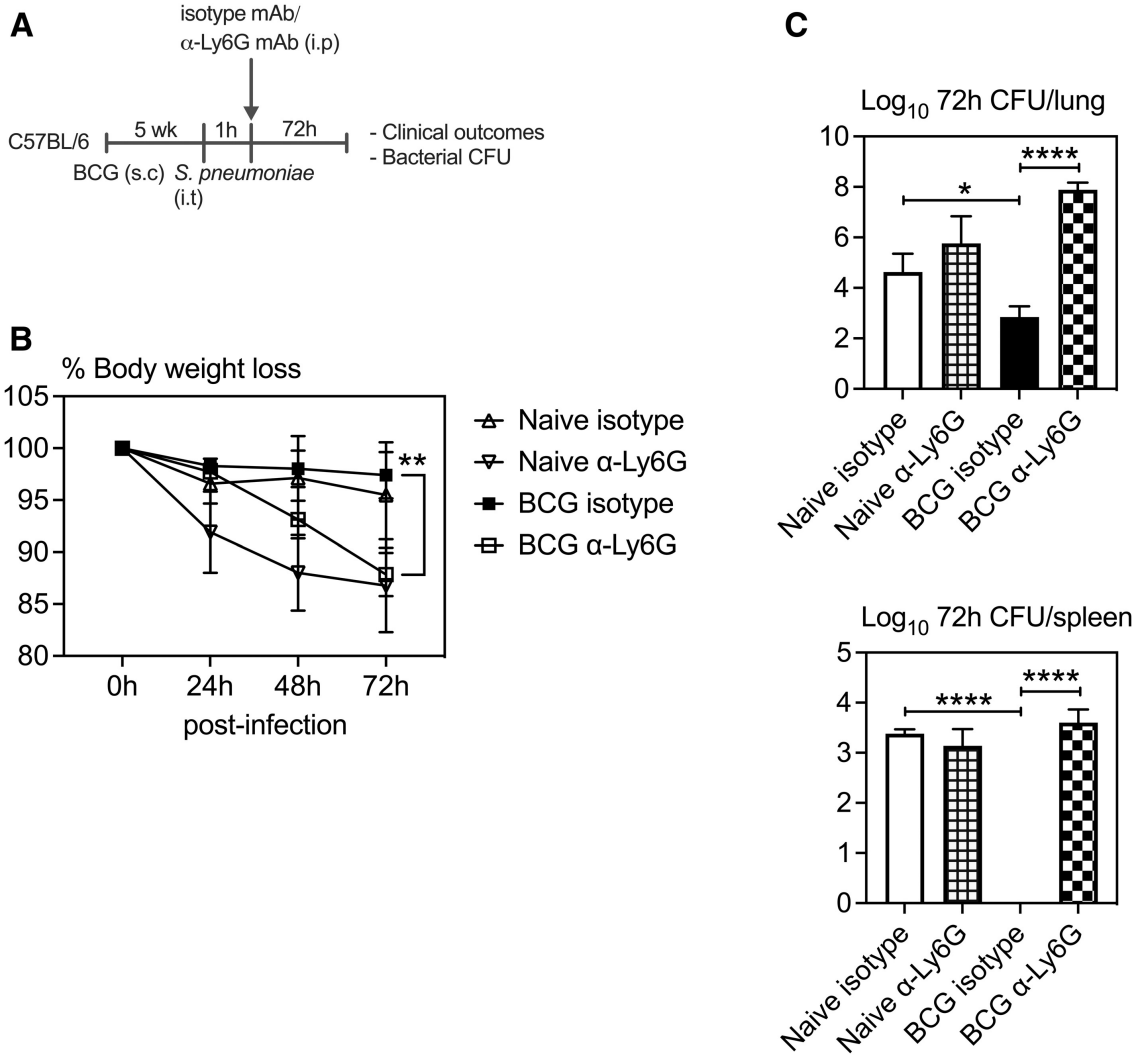
Enhanced neutrophilia plays a critical role in protection against *Streptococcus pneumoniae* infection in BCG‐vaccinated hosts Experimental schema. 5 week s.c. BCG‐vaccinated or naive mice were infected with *S. pneumoniae* i.t. and injected intraperitoneally with α‐Ly6G mAb or isotype mAb control. Mice were monitored for clinical outcomes and bacterial burden was assessed.Line graphs comparing changes in body weight following *S. pneumoniae* infection in BCG‐vaccinated and unvaccinated hosts. *N =* 4–5 mice/group.Bar graphs comparing bacterial counts (CFU) in the lung and spleen at 72 h post‐*S. pneumoniae* infection in BCG‐vaccinated and unvaccinated hosts. *N =* 4–5 mice/group. Data information: Data presented in (B and C) represent mean ± SEM of biological replicates. Statistical analysis for (B and C) were two‐way ANOVA with Sidak's multiple comparisons test, (C) was two‐tailed unpaired *t*‐test. *P < 0.05; **P < 0.01; ****P < 0.0001. Source data are available online for this figure.

Given that enhanced protection against *S. pneumoniae* infection in s.c. BCG‐vaccinated hosts was accompanied by enhanced recruitment of neutrophils to the respiratory mucosa (Fig 2B and C), we next evaluated the role of pronounced neutrophilia in such enhanced protection. To this end, using a neutrophil‐depleting mAb (α‐Ly6G) as previously described (Yao *et al*, 2018), we reduced lung neutrophilia in the lung by partially depleting neutrophils *in vivo* immediately after *S. pneumoniae* infection in naïve controls (Naïve α‐Ly6G) and BCG‐vaccinated hosts (BCG α‐Ly6G) (Fig 5A). As controls, naïve and s.c BCG‐vaccinated hosts were administered isotype antibodies (Naïve/BCG isotype). We then evaluated body weight loss as a measure of clinical outcomes and bacterial burden in the lung and spleen at 72‐h postinfection. In keeping with our earlier observation (Fig 1C), no significant changes were observed in the weight of naïve (Naïve isotype) and BCG‐vaccinated (BCG isotype) mice, treated with the isotype antibodies, at 72‐h postinfection (Fig 5B). In contrast, depletion of neutrophils in BCG‐vaccinated (BCG α‐Ly6G) mice led to significant weight loss compared with isotype antibody treated control counterparts. Similarly, neutrophil‐depleted naïve animals (Naïve α‐Ly6G) also experienced a level of weight loss compared with Naïve isotype controls. Furthermore, similar to our earlier findings (Fig 1F), BCG isotype control mice with intact neutrophilic responses better controlled *S. pneuomoniae* infection indicated by significantly smaller bacterial burden both in the lung and spleen compared with Naïve isotype controls (Fig 5C). Of importance, depletion of neutrophils in BCG‐vaccinated mice (BCG α‐Ly6G) resulted in a total loss of protection against bacterial infection both in the lung and spleen compared with their neutrophil intact counterparts (BCG isotype) (Fig 5C). While as expected, neutrophil depletion in naïve animals (Naïve α‐Ly6G) alse led to increased bacterial burden, particularly in the lung, the magnitude of such changes was much less remarkable than that in neutrophil‐depleted BCG hosts. These data together indicate that pronounced neutrophilia in subcutaneous BCG‐vaccinated hosts plays a critical role in enhanced heterologous innate protection against *S. pneumoniae* infection in the lung.

### Enhanced protection against *S. pneumoniae* infection in BCG‐vaccinated hosts is independent of circulating monocytes

**Figure 6:**
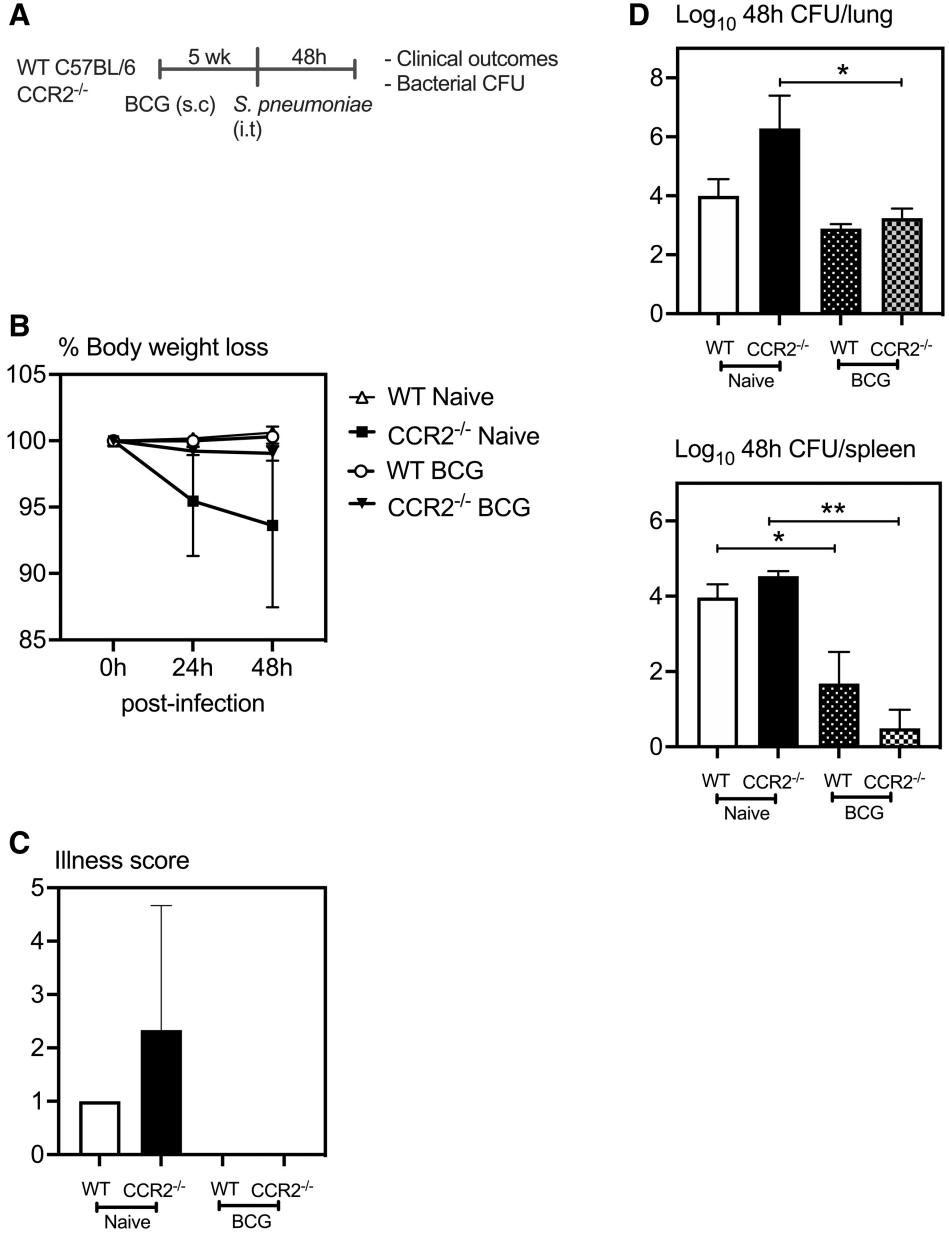
Enhanced protection against *Streptococcus pneumoniae* infection in BCG‐vaccinated hosts is independent of monocytes Experimental schema. 5 week s.c. BCG‐vaccinated or naive mice were infected with *S. pneumoniae* i.t. and monitored for clinical outcomes and bacterial CFU was assessed.Line graphs comparing changes in body weight following *S. pneumoniae* infection in BCG‐vaccinated and unvaccinated hosts. *N =* 3 mice/group.Bar graphs comparing clinical symptoms as a measure of illness score at 48 h post‐*S. pneumoniae* infection. *N =* 3 mice/group.Bar graphs comparing bacterial counts (CFU) in the lung and spleen at 48 h post‐ *S. pneumoniae* infection in BCG‐vaccinated and unvaccinated hosts. *N =* 3 mice/group. Data information: Data presented in (B–D) represent mean ± SEM of biological replicates. Statistical analysis for (B and C) was two‐way ANOVA with Sidak's multiple comparisons test, (D) was one‐way ANOVA with Tukey's multiple comparisons test. *P < 0.05; **P < 0.01. Source data are available online for this figure.

Given that i.v. BCG vaccine‐induced heterologous protection has been attributed to BCG induced trained innate immunity in circulating monocytes (Kaufmann *et al*, 2018; Cirovic *et al*, 2020), we next further addressed whether circulating monocytes could have contributed to anti‐*S. pneumoniae* innate immunity in the lung observed in our cutaneous BCG‐vaccinated model. Thus, CCR2^−/−^ mice lacking classical Ly6C^hi^ circulating monocytes (CCR2^−/−^ BCG) or wild‐type (WT) mice (WT BCG) were s.c. BCG vaccinated for 5 weeks (Fig 6A). Groups of WT (WT naïve) or CCR2^−/−^ (CCR2^−/−^ naïve) mice were left unvaccinated as controls. Mice in all groups were infected i.t. with *S. pneumoniae*. Mice were monitored daily for clinical signs and weight loss and bacterial burden in the lung and spleen were assessed by bacterial CFU assay at 48‐h postinfection, by which time ~ 70% of unvaccinated CCR2^−/−^ mice reached endpoint. Consistent with the data presented in Fig 1C, *S. pneumoniae*‐infected naïve WT mice (WT naïve), like infected BCG‐vaccinated WT mice (WT BCG), did not lose any body weight by 48‐h (Fig 6B). Nor did BCG‐vaccinated CCR2^−/−^ animals (CCR2^−/−^ BCG) (Fig 6B). In contrast, compared with unvaccinated WT mice and BCG‐vaccinated groups, unvaccinated CCR2^−/−^ (CCR2^−/−^ naïve) mice displayed both moderate loss of body weight and clinical signs (Fig 6B and C), suggesting a protective role of circulating monocytes against *S. pneumoniae* infection in unvaccinated animals. In keeping with clinical outcomes, lungs of unvaccinated CCR2^−/−^ mice also harbored increased number of bacteria compared with unvaccinated WT counterparts (Fig 6D). Of importance, BCG‐vaccinated CCR2^−/−^ animals lacking circulating monocytes were not only better protected than their unvaccinated counterparts in terms of bacterial infection in the lung and spleen but were also as well protected as BCG‐vaccinated WT hosts with circulating monocytes (Fig 6D). The above data suggest that although circulating monocytes play a critical role in innate immune protection against *S. pneumoniae* infection in the lung of unvaccinated hosts, their role can be entirely compensated for by enhanced responses of both alveolar macrophages and neutrophils in BCG‐vaccinated hosts.

## Discussion

Over the years, BCG vaccination has been shown to provide nonspecific protection against heterologous infections other than TB in humans (Netea *et al*, 2016; Pasco & Anguita, 2020; Xing *et al*, 2020). While mounting preclinical evidence attributes such protection to TII in circulating monocytes induced by i.v. BCG vaccination (Kaufmann *et al*, 2018, 2022), some studies have suggested that i.v. BCG vaccination induces much stronger trained immunity compared to cutaneous BCG vaccination (Kaufmann *et al*, 2018; Mata *et al*, 2021; Vierboom *et al*, 2021). However, we have recently reported the induction of trained immunity in lung‐resident macrophages following s.c. BCG vaccination, which provided enhanced protection against homologous *M. tb* infection (Jeyanathan *et al*, 2022). Thus, up to date, most of published preclinical studies have been at odds with human studies in that clinically or in real‐world practice, BCG vaccine is always administered via the skin. The relative paucity of clinically relevant preclinical studies on cutaneous BCG vaccination and lung TII has hindered the mechanistic understanding of cutaneous BCG vaccine‐induced TII in humans. For instance, it still remains unclear whether cutaneous BCG vaccination‐induced TII in the lung could enhance innate protection against heterologous respiratory bacterial pathogens and if so, what would be the underlying mechanisms. To fill this knowledge gap, in the current study, we have used a murine model with a low‐dose BCG inoculum to study s.c. BCG vaccine‐induced TII in the lung. Our study shows for the first time that s.c. BCG vaccine‐induced TII in the lung provides enhanced heterologous innate immune protection against pulmonary *S. pneumoniae* infection. Such enhanced protection was mediated primarily via enhanced neutrophilia in the lung, independently of centrally trained circulating monocytes. Thus, our study reveals the mechanisms by which cutaneous BCG vaccination induces TII against heterologous respiratory bacterial infections in the lung and holds implications in developing strategies to modulate immunity at a remote tissue site. It is worthwhile to point out that future investigation is needed to further understand the longevity of heterologous innate protection mediated by subcutaneous BCG‐induced TII in the lung since our current study has provided only limited information on its sustainability.

To date, preclinical studies demonstrate that only i.v. BCG vaccination is associated with TII and suggests that cutaneous BCG vaccination does not induce TII to the same extent (Kaufmann *et al*, 2018). However, our findings that lung‐resident AMs in s.c. BCG‐vaccinated hosts display heightened phagocytic and killing capacities and protect against *S. pneumoniae* independent of circulating monocytes indicates that cutaneous BCG vaccination can also remotely impact tissue‐resident AMs. This observation is further supported by a recent study that showed cutaneous BCG vaccination in mice remodels AMs in the airways and alters AM responses to *M. tb* infection via mediating a robust and dominant interferon response (preprint: Mai *et al*, 2022). Such altered functionality of AMs was long‐lasting as indicated by comparable or even elevated enhanced responses to secondary *ex vivo* stimulation at 23 weeks compared to 8 weeks post‐s.c. BCG vaccination. An independent investigation from our laboratory has revealed a gut–lung microbiota and metabolomic pathway via which s.c. BCG vaccination induces memory AMs and trained innate immunity against homologous pulmonary *M. tb* infection (Jeyanathan *et al*, 2022). The current study provides further evidence that such TII in the lung following cutaneous BCG vaccination can also mediate heterologous protection against extracellular respiratory bacterial pathogens such as *S. pneumoniae*. Interestingly, despite phenotypic and functional alterations in AMs, the gross cellularity in the airways was unaffected following cutaneous BCG vaccination, indicating minimal‐to‐no contribution of centrally trained monocytes (Jeyanathan *et al*, 2022; preprint: Mai *et al*, 2022). However, although pulmonary *S. pneumoniae* infection led to the recruitment of centrally trained monocytes to the lung in BCG‐vaccinated hosts, they did not contribute to enhanced protection as BCG‐vaccinated CCR2^−/−^ mice were protected to the same level as BCG‐vaccinated WT mice.

Although studies have demonstrated that respiratory mucosal and i.v. administered BCG vaccination provides nonspecific protection against heterologous viral and bacterial pathogens (Vierboom *et al*, 2021; Kaufmann *et al*, 2022), we have shown for the first time that cutaneous BCG vaccination provides enhanced protection against pulmonary *S. pneumoniae* infection. Although much is known regarding the mechanisms of i.v. BCG vaccine‐induced TII and nonspecific protection, more preclinical mechanistic studies are needed to better understand cutaneous BCG vaccine‐induced TII in humans. Thus far, BCG‐mediated nonspecific innate protection has been mainly attributed to circulating monocytes, NK cells and neutrophils and is associated with epigenetic and metabolic reprogramming leading to increased production of pro‐inflammatory cytokines (Kleinnijenhuis *et al*, 2012, 2014; Moorlag *et al*, 2020; Soto *et al*, 2022). It has been shown by us and others that parenteral BCG vaccination gains access to the bone marrow resulting in its training effects on hematopoietic progenitor cells (Kaufmann *et al*, 2018; Cirovic *et al*, 2020; Jeyanathan *et al*, 2022). Circulating innate immune cells, specifically neutrophils, have been shown to play a critical role in host defense against bacterial infections including *S. pneumoniae* (Taut *et al*, 2008). Our findings suggest that cutaneous BCG vaccine‐induced anti‐streptococcal TII is mediated via enhanced neutrophilia in the lung. We show that partial depletion of neutrophils in BCG‐vaccinated hosts significantly reduced protection. This is in line with our previous work in which TII induced by an adenoviral vectored vaccine augmented anti‐streptococcal protection via accelerated neutrophilia in the lung (Yao *et al*, 2018). Preclinical and clinical studies have previously reported the effects of parenteral BCG vaccination on granulopoiesis in the bone marrow and the induction of long‐lasting functional changes in neutrophils that are characteristic of TII (Brook *et al*, 2020; Moorlag *et al*, 2020). Thus, it is likely that s.c. BCG vaccination also induces granulopoiesis in our model. Based on this consideration, it cannot be ruled out that induced trained immunity in neutrophils, in concert with enhanced tissue neutrophilia and trained alveolar macrophages (Figs 2, 4, and 5), could have also contributed to enhanced protection against heterologous infection observed in our current study.

The murine model used in the current study to understand cutaneous BCG vaccine‐induced TII in the lung is unique in comparison to murine models reported in the literature, which lack clinical relevance by administering BCG via the i.v. route (Kaufmann *et al*, 2018, 2022). It is noteworthy that while live BCG is rarely found in the lung of s.c. BCG‐vaccinated mice, i.v. BCG vaccination leads to much more BCG dissemination and ongoing replication in the lung, suggesting differential impact of i.v. and s.c. route of BCG vaccination on resident AMs (Kaufmann *et al*, 2018; preprint: Mai *et al*, 2022). It has been shown that IFN‐γ plays a critical role in the generation of trained immunity in bone‐marrow‐derived myeloid cells following i.v. BCG vaccination (preprint: Mai *et al*, 2022). Similarly, T cell‐derived IFN‐γ led to training of AMs following respiratory adenoviral viral infection and trained innate immunity against *S. pneumoniae* infection (Yao *et al*, 2018). On the contrary, influenza‐induced T cell‐derived IFN‐γ resulted in AM dysfunction and impaired protection against *S. pneumoniae* infection (Verma *et al*, 2020). AM training following all these scenarios was accompanied by direct exposure of the lung microenvironment to the respective microbial agent. These together suggest that s.c. BCG vaccine‐induced TII in AMs is most likely caused by a different mechanism. Interestingly, our recent study has shown that s.c. BCG vaccination‐mediated remodeling of AMs is independent of IFN‐γ and circulating monocytes and it is rather mediated by circulating metabolites produced by gut microbiota, which was altered by s.c. BCG vaccination (Jeyanathan *et al*, 2022).

In conclusion, our study reveals that cutaneous BCG vaccine‐induced TII in the lung can improve protection against pulmonary *S. pneumoniae* infection by enhanced neutrophilia in the lung and such enhanced protection is independent of centrally trained monocytes. Such knowledge shall help design novel effective universal vaccination strategies against unrelated respiratory bacterial pathogens.

## Materials and Methods

### Mice

Wild‐type 6–8‐week‐old female C57BL/6 mice were purchased from Charles River (RRID:IMSR_CRL: 027). Chemokine (C‐C motif) receptor 2 (CCR2) knockout mice on a C57BL/6 background (B6.129S4‐*Ccr2* ^*tm1Ifc*^/J) were purchased from the Jackson Laboratory (RRID:IMSR_JAX: 004999). All mice were housed in a specific pathogen‐free level B facility at McMaster University. All experiments were conducted in accordance with the guidelines of the animal research ethics board of McMaster University.

### Mycobacterial preparation for immunization

*Mycobacterium bovis* BCG (Pasteur strain) was grown in Middlebrook 7H9 broth supplemented with Middlebrook oleic acid‐albumin‐dextrose‐catalase (OADC) enrichment (Invitrogen Life Technologies, Carlsbad, CA), 0.002% glycerol, 0.05% Tween 80 for < 10–15 days, then aliquoted and stored in −70°C until needed. Prior to each use, bacilli were washed twice with phosphate‐buffered saline (PBS) containing 0.05% Tween 80 and passed through a 27‐gauge needle 10 times to disperse clumps. Mice were vaccinated subcutaneously (s.c.) with a low‐dose 20–50,000 CFU/mouse of BCG in 100 μl of PBS. We have previously shown that this low‐dose of BCG inoculum is effective to induce strong adaptive and trained innate immunity in mice (Santosuosso *et al*, 2006; Horvath *et al*, 2012; Jeyanathan *et al*, 2022). The exact dose of vaccination was verified by tittering the inoculum using colony‐forming unit (CFU) assay.

### *Streptococcus pneumoniae* preparation for infection

A clinical isolate of *S. pneumoniae* serotype 3 (ATCC 6303; ATCC, Manassas, VA) was prepared. Briefly, the frozen bacterial stock was thawed in 37°C water bath and cultured at 37°C in 5% CO_2_ in Todd Hewitt broth (BD Biosciences, San Jose, CA, USA) to mid‐logarithmic phase, OD = 0.40–0.50. Bacteria were harvested and resuspended in cold PBS. For intratracheal (i.t.) administration, mice were infected with 10–50,000 CFU/mouse of *S. pneumoniae* in 40 μl of PBS. The stock titer and infectious dose was verified by plating 10‐fold serial dilutions on blood tryptic soy agar (BD Biosciences, San Jose, CA, USA) supplemented with 5% defibrinated sheep blood (Hemostat, Dixon, CA, USA) and 10 μg/ml neomycin (Sigma‐ Aldrich, St. Louis, MO, USA).

### Bronchoalveolar lavage and lung mononuclear cell isolation

Mice were anesthetized by isoflurane and euthanized by exsanguination 5‐wk postvaccination. Cells from bronchoalveolar lavage and lung tissue were isolated as previously described (Santosuosso *et al*, 2006). Briefly, the lung was lavaged five times to a volume of 1.35 ml of PBS by cannulating the trachea using polyethylene tubing attached to 23‐gauge needle and syringe. Following exhaustive bronchoalveolar lavage, lungs were cut into small pieces and digested in RPMI solution containing collagenase type 1 (ThermoFisher Scientific Waltham, MA, USA) for 1 h at 37°C in an agitating incubator. Single‐cell suspension was obtained by crushing the digested lung tissue through a 100 μm basket filter (BD Biosciences, San Jose, CA, USA) and subsequent lysis of RBCs by resuspending the cell pellet in Ammonium‐Chloride‐Potassium (ACK) lysing buffer for 2 min. Isolated cells were then resuspended in complete RPMI 1640 medium (RPMI 1640 supplemented with 10% FBS, 1% L‐glutamine, 10 mM HEPES, 0.5 mM Na pyruvate, 55 μM 2‐Mercaptoethanol, 0.1 mM NEAA, and with 1% penicillin/streptomycin) for flow cytometry staining. Isolated AMs from bronchoalveolar lavage were resuspended in PBS for adoptive transfer, mice were given 120,000 cells i.t. in 40 μl of PBS. Cell numbers were quantified in Turk's Blood Dilution Fluid (RICCA Chemical, Arlington, TX, USA) and counted under a microscope. Conventional BAL was collected and stored at −80°C until cytokine/chemokine measurement by Luminex.

### Immunostaining for cell phenotype characterization using flow cytometry

Cell immunostaining and flow cytometry were performed as previously described (Jeyanathan *et al*, 2017). Briefly, mononuclear cells from bronchoalveolar lavage (BAL) and the lung were plated in a U‐bottom 96‐well plate at a concentration of 20 million cells/ml in PBS. Following staining with the LIVE/DEAD™ Fixable Aqua Dead Cell Stain Kit (ThermoFisher Scientific Waltham, MA, USA) at room temperature for 20–30 min, cells were washed and blocked to avoid non‐specific staining with anti‐ CD16/CD32 (clone 2.4G2) in 0.5% BSA‐PBS (FACS buffer) for 15 min on ice and then stained with fluorochrome‐labeled mAbs for 20–30 min on ice. Fluorochrome‐labeled mAbs used for staining myeloid cells including alveolar macrophages, interstitial macrophages, monocyte‐derived macrophages, monocytes, dendritic cells, and neutrophils were anti‐CD45‐APC‐Cy7 (clone 30‐ F11, BD Biosciences, 1:400), anti‐CD11b‐PE‐Cy7 (clone M1/70, BD Biosciences, 1:400), anti‐CD11c‐APC (clone HL3, BD Biosciences, 1:200), anti‐ MHC II‐Alexa Flour 700 (clone M5/114.15.2, Thermo Fisher, 1:200), anti‐CD3‐V450 (clone 17A2, BD Biosciences, 1:200), anti‐Ly6C‐Biotin (clone HK1.4, BioLegend, 1:200), Streptavidin‐ Qdot800 (Thermo Fisher, 1:500), anti‐CD24‐BV650 (clone M1/69, BD Biosciences, 1:1,500), anti‐CD64‐PE (clone X54‐5/7.1, BioLegend, 1:100), anti‐ Ly6G‐BV605 (clone 1A8, BD Biosciences, 1:500), anti‐Siglec‐F‐PE‐ CF594 (clone E50‐2440, BD Biosciences, 1:1,500), anti‐B220 (CD45R)‐V450 (clone RA3‐6B2, BioLegend, 1:200). Immediately upon completion of the staining procedure, immunostained cells were processed according to the BD Biosciences instruction for flow cytometry and ran on a BD LSRII or BD LSRFortessa flow cytometer. Gating strategy used for the identification of macrophage subsets in the airways and lung was as the following. Live CD45+ cells were gated to remove dead cells and CD11b^+^Ly6G^+^ neutrophils. Macrophages were gated as CD64^+^CD24^−^ and further gated into macrophage subsets, monocyte‐derived macrophages (MDM) CD64^+^Ly6C^+^SiglecF^−^, interstitial macrophages (IM) CD64^+^Ly6C^−^SiglecF^−^ and alveolar macrophages (AM) CD64^+^Ly6C^−^SiglecF^+^.

### Evaluation of clinical outcomes and bacterial enumeration in tissues

Following *S. pneumoniae* infection, mice were monitored for clinical outcomes including illness scoring and body weight changes at various time points and moribund mice were terminated. Illness score was determined based on a set of parameters including appearance (ruffled, hunched), respiration (fast, slow), behavior (not moving, depressed, isolated) and body conditions. The grading system used a way of scoring with 0 for no symptoms and 1 point for the presence of each symptom mentioned above. The endpoint was defined as 15–20% loss of initial body weight. Otherwise, mice were sacrificed at indicated time points post‐*S. pneumoniae* infection for further analysis including bacterial CFU assay, flow cytometry and chemokine and cytokine analysis. For bacterial CFU assay, the lung and spleen tissues were homogenized in homogenization buffer. Serial dilutions of tissue homogenates were plated on blood agar plates and incubated overnight at 37°C in 5% CO_2_. Colonies were counted and calculated as log_10_ CFU per organ.

### *Ex vivo* bacterial stimulation, phagocytosis and killing assays

Isolated AMs from bronchoalveolar lavage were resuspended in complete RPMI medium without penicillin/ streptomycin (P/S‐free media) and plated at 1.5 × 10^5^ cells/well in 48‐well plates (100 μl/well). Cells were incubated in a 37°C 5% CO_2_ cell‐culture incubator for 1 h and washed twice with P/S‐free media. *S. pneumoniae* was grown as described above. Bacterial suspension was supplemented with 10% mouse serum (made in house from naïve and BCG C57BL/6 mice) and incubated for 30 min at 37°C and resuspended in P/S‐free media. Bacteria were supplemented to cell culture wells at a MOI = 2.5 (3.75 × 10^5^ CFU of bacteria into 1.5 × 10^5^ cells/well). Cells were then incubated for 1 h at 37°C, followed by supplementing with 500 μl complete RPMI 1640 (containing 1% penicillin/streptomycin)/well and incubated for additional 30 min to remove any cell bound bacteria. Bacterial‐stimulated AMs were lysed at 1‐, 2‐, or 12‐h postremoval of extracellular bacteria by adding 1 ml per well of autoclaved MiliQ water. Cell lysates were diluted by 1:10 serial dilutions in PBS, plated on blood agar and cultured overnight. Bacterial phagocytosis and killing were calculated based on the CFUs in the culture plates. In brief, bacterial CFU at 1‐h postbacterial stimulation was calculated as CFU/μl (phagocytosis). Bacterial CFU at 2‐h postbacterial stimulation was compared with that at 1‐h to show the percentage of viable bacteria of phagocytosed bacteria.

### *In vivo* depletion of neutrophils

To partially deplete neutrophils *in vivo*, mice were injected i.p with 100 μg of anti‐Ly6G mAb (clone 1A8 purchased from UCSF Monoclonal Antibody Core, San Fracisco) or 200 μg of isotype control mAb (clone 2A3, BioXCell, West Lebanon, NH, USA) at 1‐h post‐*S. pneumoniae* challenge.

### Quantification and statistical analysis

Detailed information regarding the statistical methods used is provided in the figure legends. All analyses were performed using the GraphPad Prism software (Version 6, GraphPad Software, La Jolla, CA, USA). Data were analyzed using the FlowJo software (Version 10; Tree Star, Ashland, OR, USA). *N* represents the number of mice analyzed per experiment. No statistical methods were used to predetermine sample sizes. Data collection and analysis were not performed blind to the conditions of the experiments. Experiments were performed independently either once or twice. Error bars indicate the standard error of the mean (SEM). A two‐tailed unpaired *t*‐test was used to compare two groups. A one‐way ANOVA with Tukey's multiple comparisons test was used to compare unpaired groups. A two‐way ANOVA with Sidak's multiple comparisons test was used to analyze multiple groups. Significance is indicated as follows: *P < 0.05; **P < 0.01; ***P < 0.001; ****P < 0.0001. Comparisons are not statistically significant unless indicated.

## Author contributions

**Alisha Kang:** Conceptualization; formal analysis; writing – original draft; project administration; writing – review and editing. **Gluke Ye:** Investigation. **Ramandeep Singh:** Investigation. **Sam Afkhami:** Investigation. **Jegarubee Bavananthasivam:** Investigation. **Xiangqian Luo:** Investigation. **Maryam Vaseghi‐Shanjani:** Investigation. **Fatemah Aleithan:** Investigation. **Anna Zganiacz:** Investigation. **Mangalakumari Jeyanathan:** Conceptualization; supervision; writing – original draft; writing – review and editing. **Zhou Xing:** Conceptualization; supervision; writing – original draft; project administration; writing – review and editing.

## Disclosure and competing interests statement

The authors declare no competing interests.

## For more information

Authors' home website: https://mirc.mcmaster.ca.

## Supporting information



Appendix

Source Data for Figure 1

Source Data for Figure 2

Source Data for Figure 3

Source Data for Figure 4

Source Data for Figure 5

Source Data for Figure 6

## Data Availability

The datasets are available in the main text or the appendix file. The flow cytometry data have been deposited in Zenodo and are publicly accessible at https://doi.org/10.5281/zenodo.7738081.
