# Supplementary material for: Subcutaneous BCG vaccination protects against streptococcal pneumonia via regulating innate immune responses in the lung
Source: EMBO Mol Med. 2023 May 9;15(7):e17084. doi: 10.15252/emmm.202217084 (PMC10331578; doi:10.15252/emmm.202217084)
Supplement: Supplementary file 1 — Appendix [file EMMM-15-e17084-s002.pdf]

Subcutaneous BCG vaccination protects against streptococcal pneumonia via regulating innate immune responses in the lung

Alisha Kang, Gluke Ye, Ramandeep Singh, Sam Afkhami, Jegarubee Bavananthasivam, Xiangqian Luo, Maryam Vaseghi-Shanjani, Fatemah Aleithan, Anna Zganiacz, Mangalakumari Jeyanathan and Zhou Xing.

Appendix table of contents:

-Appendix Figure S1: page 2

-Appendix Figure S2: page 3

Appendix Figure S1

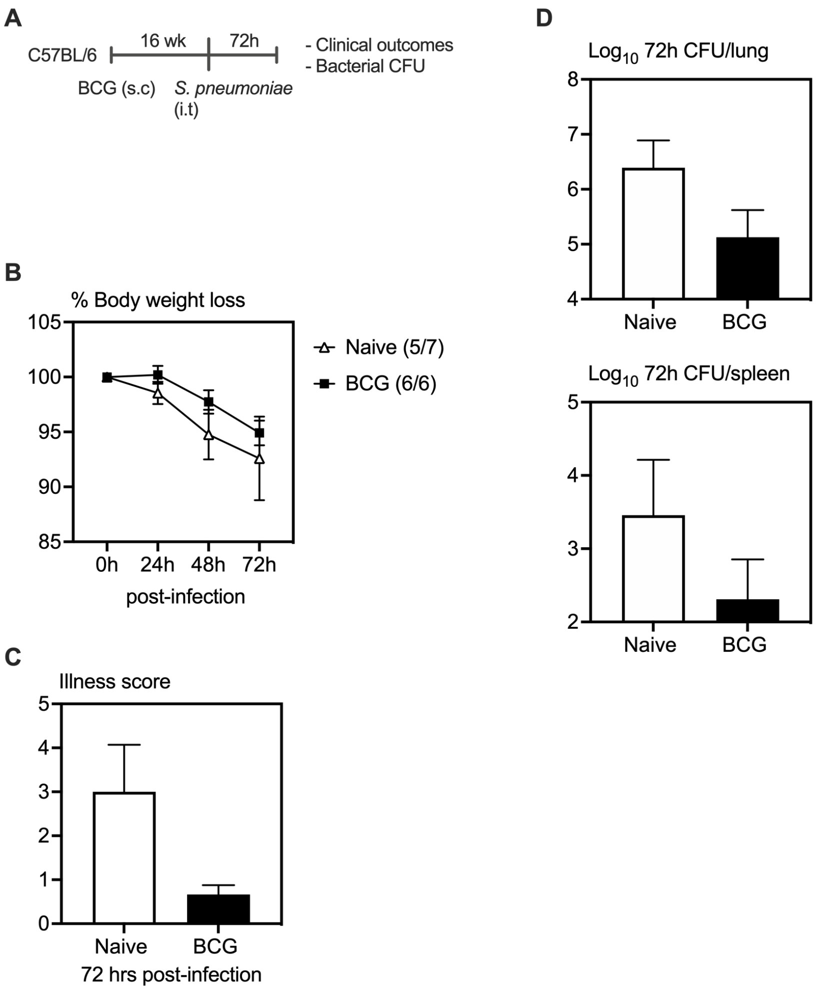

Appendix Figure S1. Enhanced protection against *S. pneumoniae* infection in BCG vaccinated hosts is long lasting

A) Experimental schema. 16 wk s.c. BCG vaccinated or unvaccinated mice were infected with *S. pneumoniae* i.t. and monitored for clinical outcomes and bacterial CFU was assessed. B) Line graphs comparing changes in body weight following *S. pneumoniae* infection in BCG vaccinated and unvaccinated hosts. N = 6 – 7 mice/group. C) Bar graphs comparing clinical symptoms as a measure of illness score at 72h post- *S. pneumoniae* infection. N = 6 – 7 mice/group. D) Bar graphs comparing bacterial counts (CFU) in the lung and spleen at 72h post-*S. pneumoniae* infection in BCG vaccinated and unvaccinated hosts. N = 6 – 7 mice/group. Data presented in (B – D) represent mean ± SEM of biological replicates. Statistical analysis for (B) was two-way ANOVA with Sidak's multiple comparisons test, (C and D) were two-tailed unpaired t-tests.

Appendix Figure S2

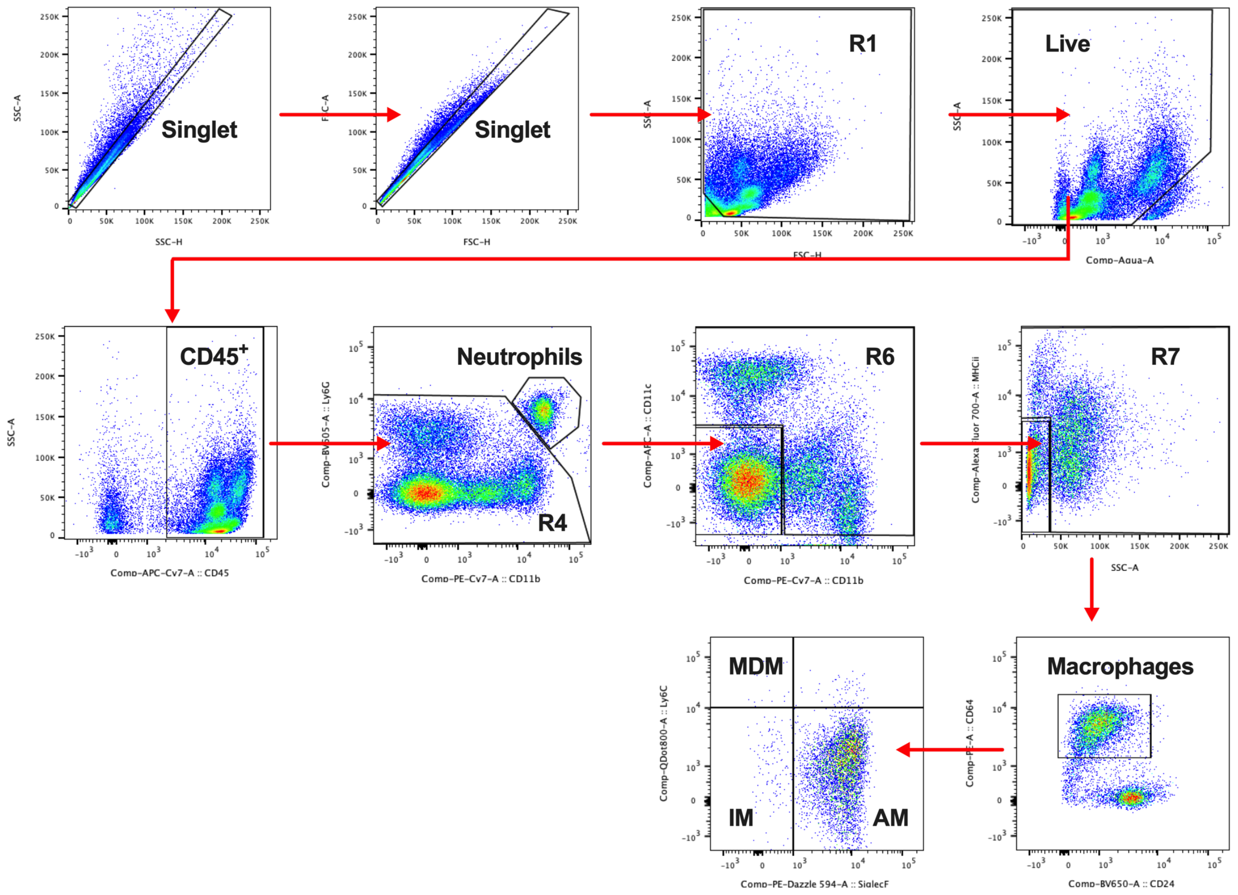

Appendix Figure S2. Flow cytometric gating strategy used to distinguish major myeloid cell populations

Gating strategy used for the identification of macrophage subsets in the airways and lung. Live CD45$^+$ cells were gated to remove dead cells and CD11b$^+$Ly6G$^+$ neutrophils. Macrophages were gated as CD64$^+$CD24$^-$ and further gated into macrophage subsets, monocyte-derived macrophages (MDM) CD64$^+$Ly6C$^+$SiglecF$^-$, interstitial macrophages (IM) CD64$^+$Ly6C$^-$SiglecF$^-$ and alveolar macrophages (AM) CD64$^+$Ly6C$^-$SiglecF$^+$.
