# Supplementary material for: Subcutaneous BCG vaccination protects against streptococcal pneumonia via regulating innate immune responses in the lung
Source: EMBO Mol Med. 2023 May 9;15(7):e17084. doi: 10.15252/emmm.202217084 (PMC10331578; doi:10.15252/emmm.202217084)
Supplement: Supplementary file 8 [file EMMM-15-e17084-s008.pdf]

EMBO Molecular Medicine

Subcutaneous BCG vaccine protects against streptococcal pneumonia via regulating lung neutrophilia

Alisha Kang, Gluke Ye, Ramandeep Singh, Sam Afkhami, Jegarubee Bavananthasivam, Xiangqian Luo, Maryam Vaseghi-Shanjani, Fatemah Aleithan, Anna Zganiacz, Mangalakumari Jeyanathan, and Zhou Xing
DOI: 10.15252/emmm.202217084

Corresponding author(s): Zhou Xing (xingz@mcmaster.ca)

Review Timeline:

Submission Date:	21st Oct 22
Editorial Decision:	7th Dec 22
Revision Received:	15th Mar 23
Editorial Decision:	6th Apr 23
Revision Received:	21st Apr 23
Accepted:	24th Apr 23

Editor: Zeljko Durdevic

Transaction Report:

(Note: With the exception of the correction of typographical or spelling errors that could be a source of ambiguity, letters and reports are not edited. Depending on transfer agreements, referee reports obtained elsewhere may or may not be included in this compilation. Referee reports are anonymous unless the Referee chooses to sign their reports.)

7th Dec 2022

Dear Prof. Xing,

Thank you for the submission of your manuscript to EMBO Molecular Medicine, and please accept my apologies for the delay in getting back to you. We have received feedback from two of the three reviewers who agreed to evaluate your manuscript. Given that referee #1 will unfortunately not be able to return his/her report in a timely manner, we prefer to make a decision now in order to avoid further delay in the process. Should referee #1 provide a report, we will send it to you, with the understanding that we will not ask for an additional revision. As you will see from the reports below, both referees recognize potential interest of the study; however, while the referee #2 is raising important but minor concerns, referee #3 raises serious concerns regarding conceptual and experimental shortcomings of the study that should be addressed in a major revision.

Addressing the reviewers' concerns in full will be necessary for further considering the manuscript in our journal, and acceptance of the manuscript will entail a second round of review. During our cross-commenting session referees agreed that point 7 from referee #3 is essential and requires additional experimentations in mice. Therefore, we think six months rather than three months would be more appropriate to provide the complete revision. If you would like to discuss further the points raised by the referees, I am available to do so via email or video. Let me know if you are interested in this option.

EMBO Molecular Medicine encourages a single round of revision only and therefore, acceptance or rejection of the manuscript will depend on the completeness of your responses included in the next, final version of the manuscript. For this reason, and to save you from any frustrations in the end, I would strongly advise against returning an incomplete revision.

We would welcome the submission of a revised version within six months for further consideration. Please let us know if you require longer to complete the revision.

I look forward to receiving your revised manuscript.

Yours sincerely,

Zeljko Durdevic

Zeljko Durdevic
Editor
EMBO Molecular Medicine

*****

When submitting your revised manuscript, please carefully review the instructions that follow below. We perform an initial quality control of all revised manuscripts before re-review; failure to include requested items will delay the evaluation of your revision.

We require:

1) A .docx formatted version of the manuscript text (including legends for main figures, EV figures and tables). Please make sure that the changes are highlighted to be clearly visible.

2) Individual production quality figure files as .eps, .tif, .jpg (one file per figure). For guidance, download the 'Figure Guide PDF': (https://www.embopress.org/page/journal/17574684/authorguide#figureformat).

3) A .docx formatted letter INCLUDING the reviewers' reports and your detailed point-by-point responses to their comments. As part of the EMBO Press transparent editorial process, the point-by-point response is part of the Review Process File (RPF), which will be published alongside your paper.

4) A complete author checklist, which you can download from our author guidelines

(https://www.embopress.org/page/journal/17574684/authorguide#submissionofrevisions). Please insert information in the checklist that is also reflected in the manuscript. The completed author checklist will also be part of the RPF.

5) Please note that all corresponding authors are required to supply an ORCID ID for their name upon submission of a revised manuscript.

6) It is mandatory to include a 'Data Availability' section after the Materials and Methods. Before submitting your revision, primary datasets produced in this study need to be deposited in an appropriate public database, and the accession numbers and database listed under 'Data Availability'. Please remember to provide a reviewer password if the datasets are not yet public (see https://www.embopress.org/page/journal/17574684/authorguide#dataavailability).

In case you have no data that requires deposition in a public database, please state so in this section. Note that the Data Availability Section is restricted to new primary data that are part of this study.

7) For data quantification: please specify the name of the statistical test used to generate error bars and P values, the number (n) of independent experiments (specify technical or biological replicates) underlying each data point and the test used to calculate p-values in each figure legend. The figure legends should contain a basic description of n, P and the test applied. Graphs must include a description of the bars and the error bars (s.d., s.e.m.). See also 'Figure Legend' guidelines: https://www.embopress.org/page/journal/17574684/authorguide#figureformat

8) At EMBO Press we ask authors to provide source data for the main manuscript figures. Our source data coordinator will contact you to discuss which figure panels we would need source data for and will also provide you with helpful tips on how to upload and organize the files.

9) Our journal encourages inclusion of *data citations in the reference list* to directly cite datasets that were re-used and obtained from public databases. Data citations in the article text are distinct from normal bibliographical citations and should directly link to the database records from which the data can be accessed. In the main text, data citations are formatted as follows: "Data ref: Smith et al, 2001" or "Data ref: NCBI Sequence Read Archive PRJNA342805, 2017". In the Reference list, data citations must be labeled with "[DATASET]". A data reference must provide the database name, accession number/identifiers and a resolvable link to the landing page from which the data can be accessed at the end of the reference. Further instructions are available at .

10) We replaced Supplementary Information with Expanded View (EV) Figures and Tables that are collapsible/expandable online. A maximum of 5 EV Figures can be typeset. EV Figures should be cited as 'Figure EV1, Figure EV2" etc... in the text and their respective legends should be included in the main text after the legends of regular figures.

- For the figures that you do NOT wish to display as Expanded View figures, they should be bundled together with their legends in a single PDF file called *Appendix*, which should start with a short Table of Content. Appendix figures should be referred to in the main text as: "Appendix Figure S1, Appendix Figure S2" etc.

- Additional Tables/Datasets should be labeled and referred to as Table EV1, Dataset EV1, etc. Legends have to be provided in a separate tab in case of .xls files. Alternatively, the legend can be supplied as a separate text file (README) and zipped together with the Table/Dataset file.

See detailed instructions here:

.

11) The paper explained: EMBO Molecular Medicine articles are accompanied by a summary of the articles to emphasize the major findings in the paper and their medical implications for the non-specialist reader. Please provide a draft summary of your article highlighting

- the medical issue you are addressing,

- the results obtained and

- their clinical impact.

This may be edited to ensure that readers understand the significance and context of the research. Please refer to any of our published articles for an example.

12) For more information: There is space at the end of each article to list relevant web links for further consultation by our readers. Could you identify some relevant ones and provide such information as well? Some examples are patient associations,

relevant databases, OMIM/proteins/genes links, author's websites, etc...

13) Author contributions: You will be asked to provide CRediT (Contributor Role Taxonomy) terms in the submission system. These replace a narrative author contribution section in the manuscript.

14) A Conflict of Interest statement should be provided in the main text.

15) Every published paper now includes a 'Synopsis' to further enhance discoverability. Synopses are displayed on the journal webpage and are freely accessible to all readers. They include a short stand first (maximum of 300 characters, including space) as well as 2-5 one-sentences bullet points that summarizes the paper. Please write the bullet points to summarize the key NEW findings. They should be designed to be complementary to the abstract - i.e. not repeat the same text. We encourage inclusion of key acronyms and quantitative information (maximum of 30 words / bullet point). Please use the passive voice. Please attach these in a separate file or send them by email, we will incorporate them accordingly.

Please also suggest a striking image or visual abstract to illustrate your article as a PNG file 550 px wide x 300-600 px high.

EMBO Molecular Medicine has a "scooping protection" policy, whereby similar findings that are published by others during review or revision are not a criterion for rejection. Should you decide to submit a revised version, I do ask that you get in touch after three months if you have not completed it, to update us on the status.

Please note: When submitting your revision you will be prompted to enter your funding and payment information. This will allow Wiley to send you a quote for the article processing charge (APC) in case of acceptance. This quote takes into account any reduction or fee waivers that you may be eligible for. Authors do not need to pay any fees before their manuscript is accepted and transferred to the publisher.

EMBO Press participates in many Publish and Read agreements that allow authors to publish Open Access with reduced/no publication charges. Check your eligibility: https://authorservices.wiley.com/author-resources/Journal-Authors/open-access/affiliation-policies-payments/index.html

***** Reviewer's comments *****

Referee #2 (Comments on Novelty/Model System for Author):

Authors describe a new model, and test an interesting hypothesis.

Referee #2 (Remarks for Author):

This is an interesting study which investigates the impact of BCG vaccination through a sub-cutaneous route on subsequent severe S. pneumoniae infection. The authors demonstrate induction of trained immunity in the lung through recruitment of neutrophils, and independent of circulating monocytes. This study fills an important gap in the literature, describing an experimental model of cutaneous BCG induction of trained immunity which is closed to BCG vaccination in humans. The experiments are well performed and the study is clearly written.

Comments:
1. While the experiments depicted in the various figures seem to be consistent with each other, the number of animals used seems to be often low (between 3 and 5 animals/group). Did the authors validate the important conclusions in independent duplicate experiments?
2. BCG has been reported to have also important effect on bone marrow progenitors of immune cells populations. Did the authors observe any effects on the bone marrow by the subcutaneous BCG administration?
3. Can the authors speculate whether the immunological effects mediated through skin or mucosae by BCG are similar or not? One would think that BCG was initially given orally in children when was first introduced in the population, and switched thereafter to intra-dermal administration.
4. Researchers from Kolmann and Amenyogbe group have shown induction of emergency granulopoiesis induced by BCG (Science Translational Medicine 2020 May 6;12(542):eaax4517.). Can the authors speculate whether this process can also play a role in the observations made here?

Referee #3 (Remarks for Author):

The manuscript presented for publication by Kang et al. describes the induction of non-specific protection induced by subcutaneous BCG against a pulmonary challenge with Streptococcus pneumoniae, and explores an immunological mechanism

underlying this heterologous benefit. Even though the topic of the manuscript is clearly of interest, considering the strong evidences of BCG-induced heterologous protection in humans, there exists a considerable number of conceptual and experimental deficiencies that makes me difficult to recommend the manuscript for publication in a journal with the quality of EMBO Molecular Medicine. Here, a list with just some of the issues found:

1. In the introduction, authors mention that other studies (references 17, 19 20) describe that s.c. BCG does not induce in mice trained immunity neither protection against heterologous infection, unlike the found by authors in this study. I would have liked to find some argument to explain this divergence between authors' results and the described in the literature. Are different the experimental settings used? mouse strains? pathogens studied?

2. What is the illness score? Not explain in the methodology section. It is based on pneumonia assessment by histology? or just weight loss and general aspect?

3. The authors aim to give response to a key question in the field as it is the mechanistic explanation behind the heterologous protection observed with BCG in humans. However the dose of BCG used is around 10 times lower than the used in clinic, without any explanation about the rationale behind this choice.

4. I would have liked to find CFUs determination from survivors to check whether BCG induces sterilizing immunity

5. Data at 16 weeks post vaccination (displayed in figure S1) are clearly overestimated. Authors state that BCG still maintains a considerable protective effect at this late timepoint. However, according to data, no statistical differences are found, just tendencies, and with regard to weigh loss (the major indicator of animal welfare) both groups drop with a similar tendency, suggesting no differences in terms of survival.

6. Flow cytometry data are displayed deficiently. No gating strategy is shown, and the definition of populations is incomplete. For instance, they define IM as Ly6C-SiglecF- population, but it is not indicated the positive markers. Probably they use CD64 to define that they are macrophages, but this is not explained in the text.

7. The results of neutrophils, which authors proposed as the major contribution of the study, are also incomplete and experiments are not well designed. Data in figure 5 do not show results from unvaccinated groups +/- antiLy6G. Therefore, authors cannot discard whether neutrophils are needed to control infection independently of BCG presence. This absence of control groups clearly weakens one of the main conclusions of the study.

8. In addition, it is highly surprising to find that in the untreated group there is a decrease of neutrophils regardless of the advance of the pneumonia and the replication of the pathogen. There is a wide consensus in the literature that the challenge model of S. pneumoniae comes with a strong induction of neutrophils both in BAL and lungs, which cause pathogenic inflammation. However, authors find that neutrophil numbers drop. I cannot find an explanation for these so contradictory results shown by the author in comparison to lots of studies (Just a couple of examples: PMID: 16988243; PMID: 25595646)

Point-by-point responses

We are very pleased of the favorable opinions of our study and appreciate the constructive comments from both Editor and reviewers. We have taken all the comments into serious consideration and performed a careful revision accordingly including conducting additional new experiments, and have addressed all the comments raised by the reviewers as detailed below. The revision has significantly strengthened our main conclusions and enhanced the clarity and quality of the manuscript.

Referee #2

Referee #2 (Remarks for Author):

This is an interesting study which investigates the impact of subcutaneous BCG vaccination on subsequent severe *S. pneumoniae* infection. The authors demonstrate induction of trained immunity in the lung through recruitment of neutrophils, and independent of circulating monocytes. This study fills an important gap in the literature, describing an experimental model of cutaneous BCG induction of trained immunity which is closed to BCG vaccination in humans. The experiments are well performed, and the study is clearly written.

Comments:

1. While the experiments depicted in the various figures seem to be consistent with each other, the number of animals used seems to be often low (between 3 and 5 animals/group). Did the authors validate the important conclusions in independent duplicate experiments?

Response: This is a relevant point. The data have now been validated fully from independent experiments for major conclusions including contribution of trained airway resident macrophages of BCG vaccinated hosts to trained innate immunity against heterologous infection (Figures 4E/F) and protective role of enhanced neutrophilia in BCG-vaccinated hosts (Figures 5B/C). Furthermore, to validate the BCG-induced trained innate immunity (TII) in lung protection in Figure 1 and in response to this comment from Reviewer, we have now also performed a new experiment and in keeping with our earlier observations, s.c. BCG vaccine-induced TII provided significantly enhanced protection against *S. pneumoniae* infection. Thus, the data presented in Figures 1B/C/D are now updated by pooling the data from two independent experiments with increased statistical power and significance, particularly for Figure 1B/D (**line 103–107**).

2. BCG has been reported to have also important effect on bone marrow progenitors of immune cells populations. Did the authors observe any effects on the bone marrow by the subcutaneous BCG administration?

Response: It has now been well established that intravenous BCG vaccination induces trained innate immunity in the blood and bone marrow monocytes (PMID: 29328912; PMID: 33521699), while the impact of subcutaneous BCG immunization on centrally trained innate immunity remained unclear. However, during the peer reviewing of our current manuscript, an independent study from us was published in *Nat Immunol* which focused on disseting the mechanisms of s.c. BCG vaccine-induced innate memory in lung-resident macrophages and revealed the critical role of the gut-lung microbial/metabolic axis (PMID: 36456739). In this published study, we did report on significantly increased myelopoiesis in the bone marrow and heightened MHC II expression on circulating monocytes of s.c. BCG-vaccinated hosts. Thus, this published study now offers a solid foundation for the novel question and objective that our current study set out to pursue, which was to investigate whether s.c. BCG vaccine-induced TII provides heterologous protection against respiratory *S. pneumoniae* infection and if so, what would be the underlying mechanisms. Since the animal model of s.c. BCG vaccination used in this published study is identical to the one used in the current manuscript, we've now included it in Introduction (**line 71–80**).

3. Can the authors speculate whether the immunological effects mediated through skin or mucosae by BCG are similar or not? One would think that BCG was initially given orally in children when was first introduced in the population and switched thereafter to intra-dermal administration.

Response: In fact, a recent study by Vierboom *et al.* reported the impact of mucosal BCG vaccination and standard intra-dermal administration ($5x10^5$ CFU-standard adult human dose) on immune alterations in rhesus macaques (PMID: 33521699). Vaccine induced adaptive immune profiling indicated higher responses in the circulation after intra-dermal over mucosal BCG delivery. On the contrary, mucosal BCG vaccination induced more prominent levels of polyfunctional antigen-specific T cell responses in the airways than the intra-dermal route. Interestingly, mucosal immunization with BCG improved induction of innate immune memory of circulating monocytes over intra-dermal BCG. However, induction of innate immune memory within the airways as measured by cytokine/chemokine production by total bronchoalveolar cells in response to *in vitro* stimulation did not differ between mucosal and intra-dermal vaccination routes. In fact, IL-1β, GM-CSF, and MIP-1α (CCL3) production by airway macrophages was decreased in mucosally vaccinated animals compared to before vaccination while production remained unchanged compared to before vaccination in intra-dermal BCG immunized animals. This is in contrary to the findings reported in mice following BCG vaccination via the subcutaneous or mucosal route with 10^6 CFU (PMID: 34559551). Intranasal BCG, but not subcutaneous BCG, induced macrophage activation in the airways. However, we have recently reported on induction of trained innate immune memory in lung resident macrophages following subcutaneous vaccination with a low dose BCG $4x10^4$ CFU in mice (PMID: 36456739). Taken together these observations suggest that the immunological effects mediated by BCG vaccination are rather different, depending on the dose, animal model and route of immunization.

4. Researchers from Kolmann and Amenyogbe group have shown induction of emergency granulopoiesis induced by BCG (Science Translational Medicine 2020 May 6;12(542):eaax4517.). Can the authors speculate whether this process can also play a role in the observations made here?

Response: This is a good point. There are preclinical and clinical studies that demonstrate the effects of parenteral BCG vaccination on granulopoiesis in the bone marrow and the induction of long-lasting functional changes in neutrophils that are characteristic of trained immunity (PMID: 32376769; PMID: 33207187). Given that subcutaneous BCG vaccination increases myelopoiesis in the bone marrow as shown in our recently published study (PMID: 36456739), it is likely that BCG vaccination also induces granulopoiesis in our current model. Such induced trained immunity in neutrophils could have potentially contributed to the functionality of pronounced lung tissue neutrophilia and enhanced protection against heterologous infection in the lung. Since we show in our manuscript that using an adoptive macrophage transfer approach, airway-resident alveolar macrophages of parenteral BCG-vaccinated hosts provided TII against heterologous *S. pneumoniae* infection in the lung (Figures 4E/F), we cannot rule out that enhanced protection in BCG-vaccinated hosts may have resulted from the conserted effort of both trained macrophages and neutrophils. We've now discussed this possibility in the discussion (**line 293-299**).

Referee #3 (Remarks for Author):

The manuscript presented for publication by Kang et al. describes the induction of non-specific protection induced by subcutaneous BCG against a pulmonary challenge with *Streptococcus pneumoniae* and explores an immunological mechanism underlying this heterologous benefit. Even though the topic of the manuscript is clearly of interest, considering the strong evidence of BCG-induced heterologous protection in humans, there exists a considerable number of conceptual and experimental deficiencies that makes me difficult to recommend the manuscript for publication in a journal with the quality of EMBO Molecular Medicine. Here, a list with just some of the issues found:

1. In the introduction, authors mention that other studies (references 17, 19 20) describe that s.c. BCG does not induce in mice trained immunity neither protection against heterologous infection, unlike the one found by authors in this study. I would have liked to find some argument to explain this divergence between authors' results and the described in the literature. Are different the experimental settings used? mouse strains? pathogens studied?

Response: This is a very good point. Indeed, relatively high BCG doses (10^6 CFU) used in these studies and the time point (4 weeks post-vaccination) examined for the induction of trained immunity are different from our current study. In our recently published independent study (PMID: 36456739), using a much lower dose of subcutaneous BCG ($4 x10^4$ CFU) vaccination, we have shown that in addition to centrally induced innate training in circulating monocytes, via a gut-lung microbial/metabolic axis/pathway, cutaneous BCG vaccination can also induce innate immune memory in lung resident macrophages at 5 weeks post-vaccination, but s.c. BCG-induced TII in the lung is time-dependent as at 2 weeks post-

vaccination no alterations in lung resident macrophages were evident. Taken together, these observations suggest that the immunological training effects mediated by s.c. BCG vaccination are both vaccine dose- and time-dependent. We've now included this consideration along with the reference in Introduction (**line 71–80**).

2. What is the illness score? Not explain in the methodology section. It is based on pneumonia assessment by histology? or just weight loss and general aspect?

Response: We should have included such details. Illness score is determined based on a set of parameters including appearance (ruffled, hunched), respiration (fast, slow), behaviour (not moving, depressed, isolated) and body condition. The grading system used was according to a way of scoring with 0 for no symptoms and 1 point for the presence of each symptom. This is now detailed in Methods (**line 621-624**).

3. The authors aim to give response to a key question in the field as it is the mechanistic explanation behind the heterologous protection observed with BCG in humans. However, the dose of BCG used is around 10 times lower than the used in clinic, without any explanation about the rationale behind this choice.

Response: We also appreciate this point. In most instances, $5x10^5$ CFU is the standard adult human dose (PMID: 33521699), which translates to 8,500 CFU/kg body weight, considering an average adult weight as 60 kg. In our current study, mice were immunized with 40-50,000 CFU which is equivalent to $2x10^6$ CFU/kg body weight. We have previously shown that this dose is effective enough to induce strong adaptive and trained innate immunity in mice (PMID: 22453678; 16861651; 36456739). Thus, in response to this comment from Reviewer, we've now described the rationale and supporting references to use this dose in Methods section (**line 560-562**).

4. I would have liked to find CFUs determination from survivors to check whether BCG induces sterilizing immunity.

Response: To address this comment, we have set up a new experiment to evaluate the bacterial burden at a later time point (7 days post-infection) (updated Figure 1F). Mice were either vaccinated s.c. with BCG or left untreated and were infected with *S. pneumoniae* at 5 weeks post-vaccination. We evaluated the bacterial burden in the lung at 7d post-infection. Our data together show that almost all of the BCG animals were well protected with either no or only a very small number of bacteria detected in the lung of s.c. BCG-vaccinated animals by day 7. If this trend were to continue, complete bacterial clearance is expected in all BCG animals in the subsequent days. In contrast, most of infected naïve animals succumbed by this time. These new data are now provided in Figure 1F and included in the results section (**line 114–120)**.

Also in response to a comment from another Reviewer, the data presented in Figures 1B/C/D are now updated by pooling the data from two independent experiments with increased statistical power and significance, particularly in Figure 1B/D. As a result, collectively the conclusion that s.c. BCG vaccine-induced TII in the lung enhances anti-streptococcal innate protection, is further strengthened.

5. Data at 16 weeks post vaccination (displayed in figure S1) are clearly overestimated. Authors state that BCG still maintains a considerable protective effect at this late timepoint. However, according to data, no statistical differences are found, just tendencies, and with regard to weigh loss (the major indicator of animal welfare) both groups drop with a similar tendency, suggesting no differences in terms of survival.

Response: In response, the conclusions drawn from the 16 weeks post-BCG experiment (Appendix Figure S1) have been amended as follows to better reflect the data presented. "Although both groups lost body weight to a similar extent, illness related symptoms tend to be lower in BCG-vaccinated hosts (Appendix Figure S1B/C). In keeping with reduced illness related symptoms, bacterial burden in the lung and spleen of BCG-vaccinated hosts was ~ one log less compared to naïve counterparts (Appendix Figure S1D)" (**line 122-125**).

6. Flow cytometry data are displayed deficiently. No gating strategy is shown, and the definition of populations is incomplete. For instance, they define IM as Ly6C-SiglecF- population, but it is not indicated the positive markers. Probably they use CD64 to define that they are macrophages, but this is not explained in the text.

Response: In response, a detailed gating strategy demonstrating how we distinguished macrophage subpopulations has now been provided in the new supplementary figure (Appendix Figure S2). Indeed, CD64 surface marker was used to identify macrophages before categorizing them into subsets using SiglecF and Ly6C surface markers. This information is now included in Methods (**line 611-616**).

7. The results of neutrophils, which authors proposed as the major contribution of the study, are also incomplete and experiments are not well designed. Data in figure 5 do not show results from unvaccinated groups +/- antiLy6G. Therefore, authors cannot discard whether neutrophils are needed to control infection independently of BCG presence. This absence of control groups clearly weakens one of the main conclusions of the study.

Response: We agree with the reviewer that including unvaccinated groups would strengthen the conclusion regarding the role of pronounced neutrophilia in enhanced protection in s.c. BCG hosts. In response, we have set up a new experiment to further assess the role of pronounced neutrophilia in enhanced protection against heterologous *S. pneumoniae* infection in BCG-vaccinated hosts. Mice were either vaccinated s.c. with BCG or left untreated and were infected with *S. pneumoniae* at 5 weeks post-vaccination. Immediately after infection, naïve controls and BCG vaccinated animals were either administered an isotype mAb or neutrophil-depleting mAb (α-Ly6G) intraperitoneally (Figure 5A). We then evaluated body weight loss as a measure of clinical outcomes over the course of 72 hours and assessed bacterial burden in the lung and spleen at 72h post-infection.

Our data have independently reaffirmed the conclusions that s.c. BCG vaccination significantly reduced bacterial infection (CFU) both in the lung and spleen (Naïve isotype vs BCG isotype) and that significant reduction of tissue neutrophilia via Ly6G-mediated PMN depletion led to complete loss of BCG-enhanced protection in both body weight changes and tissue bacterial CFU (BCG isotype vs BCG a-Ly6G) (Figure 5B/C), indicating a critical role of pronounced neutrophilic responses in s.c. BCG vaccine-enhanced innate protection against streptococcal infection. On the other hand, while as expected, PMN depletion in naïve animals (naïve a-Ly6G) also led to somewhat worsened clinical outcomes, similar to BCG a-Ly6G animals (Figure 5B), with increased bacterial CFU, particularly in the lung (Figure 5C), the magnitude of impaired protection against bacterial CFU seen in naïve a-Ly6G group was not as remarkable as that in BCG a-Ly6G hosts, further suggesting heightened tissue neutrophilia in s.c. BCG-vaccinated hosts to be a critical cellular mechanism. Of note, in keeping with our earlier observations (Figure 1C), no significant changes were observed in body weight of naïve (Naïve isotype) and BCG-vaccinated (BCG isotype) mice treated with the isotype antibodies, at the timepoint of 72h post-infection. These new data are now provided in revised Figure 5 and discribed in the results section (**line 185-205**).

8. In addition, it is highly surprising to find that in the untreated group there is a decrease of neutrophils regardless of the advance of the pneumonia and the replication of the pathogen. There is a wide consensus in the literature that the challenge model of S. pneumoniae comes with a strong induction of neutrophils both in BAL and lungs, which cause pathogenic inflammation. However, authors find that neutrophil numbers drop. I cannot find an explanation for these so contradictory results shown by the author in comparison to lots of studies (Just a couple of examples: PMID: 16988243; PMID: 25595646)

Response: This is a good point. In our model, we observed a different kinetics in neutrophilic responses between the airways and lung tissue of naïve animals. The number of neutrophils was increased in the airways of naïve controls at 18h post-infection and was somewhat maintained, though a bit lower than at 18h, up to 36h post-infection (Figures 2B/C) and these findings are in line with a study that reported low neutrophil responses in the bronchoalveolar lavage of young mice at 24h post *S. pneumoniae* infection (PMID: 25595646). However, in naïve controls, neutrophil counts in the lung were higher at 36h post-infection than at 18h (Figure 3B). Our study examined neutrophil responses at early timepoints following *S. pneumoniae* infection whereas, many other studies examined neutrophil responses at later timepoints, 24h or after, and did not report increased neutrophilia until after 48h post-infection (PMID: 16988243).

6th Apr 2023

Dear Prof. Xing,

Thank you for the submission of your revised manuscript to EMBO Molecular Medicine. I am pleased to inform you that we will be able to accept your manuscript pending the following final amendments:

1) Please address the referee #3 concerns by discussing in the manuscript text the limitations of the study regarding the clinical/translational implications.
2) In the main manuscript file, please do the following:
- Correct/answer the track changes suggested by our data editors by working from the attached document.
- Add keywords to main manuscript file.
- Add "The Paper Explained" to the main manuscript file.
- In M&M, the statistical paragraph should reflect all information that you have filled in the Authors Checklist, especially regarding randomization, blinding, replication.
- Please rename "Competing Interest" to "Disclosure Statement & Competing Interests". We updated our journal's competing interests policy in January 2022 and request authors to consider both actual and perceived competing interests. Please review the policy https://www.embopress.org/competing-interests and update your competing interests if necessary.
- Author contributions: Please remove it from the manuscript and specify author contributions in our submission system. CRediT has replaced the traditional author contributions section because it offers a systematic machine-readable author contributions format that allows for more effective research assessment. You are encouraged to use the free text boxes beneath each contributing author's name to add specific details on the author's contribution. More information is available in our guide to authors:
https://www.embopress.org/page/journal/17574684/authorguide#authorshipguidelines
- Add data availability statement. If no data are deposited in public repositories, please add the sentence: "This study includes no data deposited in external repositories".
Please check "Author Guidelines" for more information.
https://www.embopress.org/page/journal/17574684/authorguide#availabilityofpublishedmaterial
- Please correct the reference citation in the text and reference list. In the text of the manuscript, a reference should be cited by author and year of publication. Include a space between a word and the opening parenthesis of the reference that follows. In the reference list, citations should be listed in alphabetical order. Where there are more than 10 authors on a paper, 10 will be listed, followed by "et al.". Please check "Author Guidelines" for more information.
https://www.embopress.org/page/journal/17574684/authorguide#referencesformat
3) Appendix: Please add table of content on the first page and rename the figures to Appendix Figure S1 and S2 (as it is already in the main text).
4) Funding: Please make sure that information about all sources of funding are complete in both our submission system and in the manuscript. Currently, information about National Sanitarium Association of Canada, Canadian Foundation of Innovation are missing in our system.
5) Synopsis:
- Synopsis image: Please provide a high-resolution jpeg file 550 px-wide x (250-400)-px high.
- Synopsis text: Please upload it as separate .doc file.
- Please check your synopsis text and image before submission with your revised manuscript. Please be aware that in the proof stage minor corrections only are allowed (e.g., typos).
6) For more information: This space should be used to list relevant web links for further consultation by our readers. Could you identify some relevant ones and provide such information as well? Some examples are patient associations, relevant databases, OMIM/proteins/genes links, author's websites, etc...
7) Source data: Please upload source data as clearly labelled one (zipped) file per figure.
8) As part of the EMBO Publications transparent editorial process initiative (see our Editorial at http://embomolmed.embopress.org/content/2/9/329), EMBO Molecular Medicine will publish online a Review Process File (RPF) to accompany accepted manuscripts. This file will be published in conjunction with your paper and will include the anonymous referee reports, your point-by-point response and all pertinent correspondence relating to the manuscript. Let us know whether you agree with the publication of the RPF and as here, if you want to remove or not any figures from it prior to publication. Please note that the Authors checklist will be published at the end of the RPF.
9) Please provide a point-by-point letter INCLUDING my comments as well as the reviewer's reports and your detailed responses (as Word file).

I look forward to reading a new revised version of your manuscript as soon as possible.

Yours sincerely,

Zeljko Durdevic

Zeljko Durdevic
Editor
EMBO Molecular Medicine

*** Instructions to submit your revised manuscript ***

*** PLEASE NOTE *** As part of the EMBO Publications transparent editorial process initiative (see our Editorial at https://www.embopress.org/doi/pdf/10.1002/emmm.201000094), EMBO Molecular Medicine will publish online a Review Process File to accompany accepted manuscripts.

In the event of acceptance, this file will be published in conjunction with your paper and will include the anonymous referee reports, your point-by-point response and all pertinent correspondence relating to the manuscript. If you do NOT want this file to be published, please inform the editorial office at contact@embomolmed.org.

When submitting your revised manuscript, please include:

1) a .docx formatted version of the manuscript text (including Figure legends and tables)

2) Separate figure files*

3) supplemental information as Expanded View and/or Appendix. Please carefully check the authors guidelines for formatting Expanded view and Appendix figures and tables at https://www.embopress.org/page/journal/17574684/authorguide#expandedview

4) a letter INCLUDING the reviewer's reports and your detailed responses to their comments (as Word file).

5) The paper explained: EMBO Molecular Medicine articles are accompanied by a summary of the articles to emphasize the major findings in the paper and their medical implications for the non-specialist reader. Please provide a draft summary of your article highlighting
- the medical issue you are addressing,
- the results obtained and
- their clinical impact.
This may be edited to ensure that readers understand the significance and context of the research.
Please refer to any of our published articles for an example.

6) For more information: There is space at the end of each article to list relevant web links for further consultation by our readers. Could you identify some relevant ones and provide such information as well? Some examples are patient associations, relevant databases, OMIM/proteins/genes links, author's websites, etc...

7) Author contributions: the contribution of every author must be detailed in a separate section.

8) EMBO Molecular Medicine now requires a complete author checklist (https://www.embopress.org/page/journal/17574684/authorguide) to be submitted with all revised manuscripts. Please use the checklist as guideline for the sort of information we need WITHIN the manuscript. The checklist should only be filled with page numbers were the information can be found. This is particularly important for animal reporting, antibody dilutions (missing) and exact values and n that should be indicted instead of a range.

9) Every published paper now includes a 'Synopsis' to further enhance discoverability. Synopses are displayed on the journal webpage and are freely accessible to all readers. They include a short stand first (maximum of 300 characters, including space) as well as 2-5 one sentence bullet points that summarise the paper. Please write the bullet points to summarise the key NEW findings. They should be designed to be complementary to the abstract - i.e. not repeat the same text. We encourage inclusion of key acronyms and quantitative information (maximum of 30 words / bullet point). Please use the passive voice. Please attach these in a separate file or send them by email, we will incorporate them accordingly.

You are also welcome to suggest a striking image or visual abstract to illustrate your article. If you do please provide a jpeg file 550 px-wide x 400-px high.

10) A Conflict of Interest statement should be provided in the main text

11) Please note that we now mandate that all corresponding authors list an ORCID digital identifier. This takes <90 seconds to complete. We encourage all authors to supply an ORCID identifier, which will be linked to their name for unambiguous name identification.

Currently, our records indicate that the ORCID for your account is 0000-0001-7856-0663.

Please click the link below to modify this ORCID:
Link Not Available

12) The system will prompt you to fill in your funding and payment information. This will allow Wiley to send you a quote for the article processing charge (APC) in case of acceptance. This quote takes into account any reduction or fee waivers that you may be eligible for. Authors do not need to pay any fees before their manuscript is accepted and transferred to our publisher.

*Additional important information regarding Figures

Each figure should be given in a separate file and should have the following resolution:
Graphs 800-1,200 DPI
Photos 400-800 DPI
Colour (only CMYK) 300-400 DPI"

Figures are not edited by the production team. All lettering should be the same size and style; figure panels should be indicated by capital letters (A, B, C etc). Gridlines are not allowed except for log plots. Figures should be numbered in the order of their appearance in the text with Arabic numerals. Each Figure must have a separate legend and a caption is needed for each panel.

*Additional important information regarding figures and illustrations can be found at https://bit.ly/EMBOPressFigurePreparationGuideline. See also figure legend preparation guidelines: https://www.embopress.org/page/journal/17574684/authorguide#figureformat

The system will prompt you to fill in your funding and payment information. This will allow Wiley to send you a quote for the article processing charge (APC) in case of acceptance. This quote takes into account any reduction or fee waivers that you may be eligible for. Authors do not need to pay any fees before their manuscript is accepted and transferred to our publisher.

***** Reviewer's comments *****

Referee #2 (Comments on Novelty/Model System for Author):

This is a relevant study that fills an important knowledge gap. The experiments are well performed.

Referee #2 (Remarks for Author):

The authors responded adequately to my suggestions.

Referee #3 (Remarks for Author):

I appreciate that the authors have done a big effort to answer many of the big concerns I expressed in my previous revision of the study. However I have some concerns that make me doubt about the suitability of this manuscript to be published in a journal with the quality of EMBO Molecular Medicine. Authors have conducted experiments to analyze bacterial CFUs at 7 days (figure 1) as well as to include an unvaccinated control group with the neutrophil-neutralizing antibody against Ly6G in the figure 5. However, even though the new results obtained support the initial conclusions of the study, I have some concerns that make me consider that the impact of the study, in terms of a possible applicability into the clinics, is low. First one is that, except for figure 1, all the experiments are monitored only until 72 hours, so this means that the conclusions obtained are valid for an early phase of the infection, but not for the final outcome, which it is the critical part to make the results more impactful, both in the context of the topic of the study and the scope of this journal that pursues translational research. In addition, the new data in figure 1, CFUs are only analyzed in lungs. I thinks authors should have used this new experiment to extend CFUs determination

to other organs including spleen, and especially blood, to assess bacterial dissemination and sepsis.
With regard to the data of protection at long-term after vaccination (shown in sup material), it is only assessed until day 3. Again, evaluation of protection in terms of survival should have been the way to make these results more impactful. However, looking at the tendencies in loss of weigh it might be anticipated that the protection against survival might be worse than the observed at short-term in figure 1. The strong waning of protection of the vaccine with time clearly dampen a possible application of the use of BCG against non-specific respiratory bacterial infections.

EMM –17084-V2 point-by-point responses

April 19th, 2023

Dear Dr. Zeljko Durdevic,

We are very pleased that our manuscript is acceptable upon final amendments. Please see the point-by-point responses below:

1) *Please address the referee #3 concerns by discussing in the manuscript text the limitations of the study regarding the clinical/translational implications.*
Response: We have discussed this point in the discussion (**line 301-303**).

2) *In the main manuscript file, please do the following*:
- *Correct/answer the track changes suggested by our data editors by working from the attached document*.
Response: The track changes listed in the figure legends have been addressed and reference citation in the text and the reference list have been corrected to the EMM citation style.

- *Add keywords to main manuscript file.*
Response: Five Keywords have been added to the main manuscript file under the abstract.

- *Add "The Paper Explained" to the main manuscript file*.
Response: The paper explained has now been added to the main manuscript file.

- *In M&M, the statistical paragraph should reflect all information that you have filled in the Authors Checklist, especially regarding randomization, blinding, replication.*
Response: The quantification and statistical analysis paragraph in the M&M has been updated and includes what has been filled in the author checklist. We have included statements to address randomization, blinding and replication. Please see the updated statistical paragraph below:

- *Please rename "Competing Interest" to "Disclosure Statement & Competing Interests". We updated our journal's competing interests policy in January 2022 and request authors to consider both actual and perceived competing interests.*
Response: Competing interest has been changed to "Disclosure statement & competing interest".

- *Author contributions: Please remove it from the manuscript and specify author contributions in our submission system. CRediT has replaced the traditional author contributions section because it offers a systematic machine-readable author contributions format that allows for more effective research assessment. You are encouraged to use the free text boxes beneath each contributing author's name to add specific details on the author's contribution.*
Response: Author contributions has been removed from the manuscript file and is provided in the online submission system.

- *Add data availability statement. If no data are deposited in public repositories, please add the sentence: "This study includes no data deposited in external repositories".*

Response: The data availability section has been updated as "The datasets are available in the main text or the appendix file. The flow cytometry data has been deposited in Zenodo and are publicly accessible at https://zenodo.org".

- Please correct the reference citation in the text and reference list. In the text of the manuscript, a reference should be cited by author and year of publication. Include a space between a word and the opening parenthesis of the reference that follows. In the reference list, citations should be listed in alphabetical order. Where there are more than 10 authors on a paper, 10 will be listed, followed by "et al.".

Response: Reference citation in the text and the reference list have been amended as per journal requirement.

3) *Appendix: Please add table of content on the first page and rename the figures to Appendix Figure S1 and S2 (as it is already in the main text).*

Response: The appendix has been updated and includes a table of contents on the first page, figures have been renamed to Appendix Figure S1 and Appendix Figure S2.

4) *Funding: Please make sure that information about all sources of funding are complete in both our submission system and in the manuscript. Currently, information about National Sanitarium Association of Canada, Canadian Foundation of Innovation are missing in our system.*

Response: done

5) *Synopsis:*

- Synopsis image: Please provide a high-resolution jpeg file 550 px-wide x (250-400)-px high.

- Synopsis text: Please upload it as separate .doc file.

- Please check your synopsis text and image before submission with your revised manuscript. Please be aware that in the proof stage minor corrections only are allowed (e.g., typos).

Response: The synopsis image file type and size have been updated as per journal requirement. The synopsis has been added as a separate doc file. We confirm that the synopsis text and image have been checked for accuracy.

6) *For more information: This space should be used to list relevant web links for further consultation by our readers. Could you identify some relevant ones and provide such information as well? Some examples are patient associations, relevant databases, OMIM/proteins/genes links, author's websites, etc...*

Response: We have now included a new section "For more information" and provided the link for the author's home website for the McMaster Immunology Research Center.

7) *Source data: Please upload source data as clearly labelled one (zipped) file per figure.*

Response: Source data has been labelled as one zipped file per figure, labelled as Figure 1 source data, Figure 2 source data etc.

8) *As part of the EMBO Publications transparent editorial process initiative (see our Editorial at http://embomolmed.embopress.org/content/2/9/329), EMBO Molecular Medicine will publish online a Review Process File (RPF) to accompany accepted manuscripts. This file will be published in conjunction with your paper and will include the anonymous referee reports, your point-by-point*

response and all pertinent correspondence relating to the manuscript. Let us know whether you agree with the publication of the RPF and as here, if you want to remove or not any figures from it prior to publication. Please note that the Authors checklist will be published at the end of the RPF.
Response: We agree with the publication of the RPF.

9) *Please provide a point-by-point letter INCLUDING my comments as well as the reviewer's reports and your detailed responses (as Word file).*
Response: A point-by-point letter has been included.

Reviewer's comments

Referee #2 (Comments on Novelty/Model System for Author):

This is a relevant study that fills an important knowledge gap. The experiments are well performed.

Referee #2 (Remarks for Author):

The authors responded adequately to my suggestions.

Referee #3 (Remarks for Author):

All the experiments are monitored only until 72 hours, so this means that the conclusions obtained are valid for an early phase of the infection, but not for the final outcome, which it is the critical part to make the results more impactful, both in the context of the topic of the study and the scope of this journal that pursues translational research.
Response: In Figure 1 where we provide comprehensive information on subcutaneous BCG vaccine-mediated heterologous protection in the lung, we followed up to 7 days for survival rates, body weight changes, and lung bacterial CFU.

In addition, the new data in figure 1, CFUs are only analyzed in lungs. I think authors should have used this new experiment to extend CFUs determination to other organs including spleen, and especially blood, to assess bacterial dissemination and sepsis.
Response: Based on the data presented in Figure 1F, we observed only very few CFUs in the lung at 7d post-infection in BCG vaccinated hosts, and thus, we we do not expect to capture bacteria in other organs at this timepoint in BCG vaccinated hosts.

Regarding the data of protection at long-term after vaccination (shown in sup material), it is only assessed until day 3. Again, evaluation of protection in terms of survival should have been the way to make these results more impactful. However, looking at the tendencies in loss of weigh it might be anticipated that the protection against survival might be worse than the observed at short-term in figure 1. The strong waning of protection of the vaccine with time clearly dampen a possible application of the use of BCG against non-specific respiratory bacterial infections.
Response: We agree that the information we provided in supplemental materials is rather limited in support of a long-term protective effect after vaccination. As suggested by Editor, we have now discussed this limitation and suggested future investigation for further understanding (**line 301 – 303**).

24th Apr 2023

Dear Prof. Xing,

We are pleased to inform you that your manuscript is accepted for publication and is now being sent to our publisher to be included in the next available issue of EMBO Molecular Medicine.

Please read below for additional IMPORTANT information regarding your article, its publication and the production process.

Congratulations on your interesting work,

Zeljko Durdevic

Zeljko Durdevic
Editor
EMBO Molecular Medicine

Follow us on Twitter @EmboMolMed
Sign up for eTOCs at embopress.org/alertsfeeds

*** *** *** IMPORTANT INFORMATION *** *** ***

SPEED OF PUBLICATION
The journal aims for rapid publication of papers, using using the advance online publication "Early View" to expedite the process: A properly copy-edited and formatted version will be published as "Early View" after the proofs have been corrected. Please help the Editors and publisher avoid delays by providing e-mail address(es), telephone and fax numbers at which author(s) can be contacted.

Should you be planning a Press Release on your article, please get in contact with embomolmed@wiley.com as early as possible, in order to coordinate publication and release dates.

LICENSE AND PAYMENT:

All articles published in EMBO Molecular Medicine are fully open access: immediately and freely available to read, download and share.

EMBO Molecular Medicine charges an article processing charge (APC) to cover the publication costs. You, as the corresponding author for this manuscript, should have already received a quote with the article processing fee separately. Please let us know in case this quote has not been received.

Once your article is at Wiley for editorial production you will receive an email from Wiley's Author Services system, which will ask you to log in and will present you with the publication license form for completion. Within the same system the publication fee can be paid by credit card, an invoice, pro forma invoice or purchase order can be requested.

Payment of the publication charge and the signed Open Access Agreement form must be received before the article can be published online.

PROOFS

You will receive the proofs by e-mail approximately 2 weeks after all relevant files have been sent o our Production Office. Please return them within 48 hours and if there should be any problems, please contact the production office at embopressproduction@wiley.com.

Please inform us if there is likely to be any difficulty in reaching you at the above address at that time. Failure to meet our

deadlines may result in a delay of publication.

All further communications concerning your paper proofs should quote reference number EMM-2022-17084-V3 and be directed to the production office at embopressproduction@wiley.com.

Thank you,

Zeljko Durdevic
Editor
EMBO Molecular Medicine

EMBO Press Author Checklist

Corresponding Author Name: Alisha Kang	
Journal Submitted to: EMBO Molecular Medicine	
Manuscript Number: 17084	

USEFUL LINKS FOR COMPLETING THIS FORM
The EMBO Journal - Author Guidelines
EMBO Reports - Author Guidelines
Molecular Systems Biology - Author Guidelines
EMBO Molecular Medicine - Author Guidelines

Reporting Checklist for Life Science Articles (updated January
This checklist is adapted from Materials Design Analysis Reporting (MDAR) Checklist for Authors. MDAR establishes a minimum set of requirements in transparent reporting in the life sciences (see Statement of Task: 10.31222/osf.io/9sm4x). Please follow the journal's guidelines in preparing your

Please note that a copy of this checklist will be published alongside your article.

Abridged guidelines for figures
1. Data
The data shown in figures should satisfy the following conditions:
- the data were obtained and processed according to the field's best practice and are presented to reflect the results of the experiments in an accurate and unbiased manner.
- ideally, figure panels should include only measurements that are directly comparable to each other and obtained with the same assay.
- plots include clearly labeled error bars for independent experiments and sample sizes. Unless justified, error bars should not be shown for technical
- if n<5, the individual data points from each experiment should be plotted. Any statistical test employed should be justified.
- Source Data should be included to report the data underlying figures according to the guidelines set out in the authorship guidelines on Data

2. Captions
Each figure caption should contain the following information, for each panel where they are relevant:
- a specification of the experimental system investigated (eg cell line, species name).
- the assay(s) and method(s) used to carry out the reported observations and measurements.
- an explicit mention of the biological and chemical entity(ies) that are being measured.
- an explicit mention of the biological and chemical entity(ies) that are altered/varied/perturbed in a controlled manner.
- the exact sample size (n) for each experimental group/condition, given as a number, not a range;
- a description of the sample collection allowing the reader to understand whether the samples represent technical or biological replicates (including how many animals, litters, cultures, etc.).
- a statement of how many times the experiment shown was independently replicated in the laboratory.
- definitions of statistical methods and measures:
  - common tests, such as t-test (please specify whether paired vs. unpaired), simple χ2 tests, Wilcoxon and Mann-Whitney tests, can be unambiguously identified by name only, but more complex techniques should be described in the methods section;
  - are tests one-sided or two-sided?
  - are there adjustments for multiple comparisons?
  - exact statistical test results, e.g., P values = x but not P values < x;
  - definition of 'center values' as median or average;
  - definition of error bars as s.d. or s.e.m.

Please complete ALL of the questions below.
Select "Not Applicable" only when the requested information is not relevant for your study.

Materials

Newly Created Materials	Information included in the manuscript?	In which section is the information available? (Reagents and Tools Table, Materials and Methods, Figures, Data Availability Section)
New materials and reagents need to be available; do any restrictions apply?	Not Applicable	

Antibodies	Information included in the manuscript?	In which section is the information available? (Reagents and Tools Table, Materials and Methods, Figures, Data Availability Section)
For **antibodies** provide the following information:<br>- Commercial antibodies: RRID (if possible) or supplier name, catalogue number and or/clone number<br>- Non-commercial: RRID or citation	Yes	Materials and methods (p.26-30)

DNA and RNA sequences	Information included in the manuscript?	In which section is the information available? (Reagents and Tools Table, Materials and Methods, Figures, Data Availability Section)
**Short novel DNA or RNA including primers, probes**: provide the sequences.	Not Applicable	

Cell materials	Information included in the manuscript?	In which section is the information available? (Reagents and Tools Table, Materials and Methods, Figures, Data Availability Section)
**Cell lines:** Provide species information, strain. Provide accession number in repository **OR** supplier name, catalog number, clone number, and/**OR** RRID.	Not Applicable	
**Primary cultures:** Provide species, strain, sex of origin, genetic modification status.	Yes	Materials and methods
Report if the cell lines were recently **authenticated** (e.g., by STR profiling) and tested for mycoplasma contamination.	Not Applicable	

Experimental animals	Information included in the manuscript?	In which section is the information available? (Reagents and Tools Table, Materials and Methods, Figures, Data Availability Section)
**Laboratory animals or Model organisms:** Provide species, strain, sex, age, genetic modification status. Provide accession number in repository **OR** supplier name, catalog number, clone number, **OR** RRID.	Yes	Materials and methods (p.26)
**Animal observed in or captured from the field:** Provide species, sex, and age where possible.	Not Applicable	
Please detail **housing and husbandry conditions**.	Yes	Materials and methods (p.26)

Plants and microbes	Information included in the manuscript?	In which section is the information available? (Reagents and Tools Table, Materials and Methods, Figures, Data Availability Section)
**Plants:** provide species and strain, ecotype and cultivar where relevant, unique accession number if available, and source (including location for collected wild specimens).	Not Applicable	
**Microbes:** provide species and strain, unique accession number if available, and source.	Yes	Materials and methods

Human research participants	Information included in the manuscript?	In which section is the information available? (Reagents and Tools Table, Materials and Methods, Figures, Data Availability Section)
If collected and within the bounds of privacy constraints report on age, sex and gender or ethnicity for all study participants.	Not Applicable	

Core facilities	Information included in the manuscript?	In which section is the information available? (Reagents and Tools Table, Materials and Methods, Figures, Data Availability Section)
If your work benefited from core facilities, was their service mentioned in the acknowledgments section?	Not Applicable	

Design

Study protocol	Information included in the manuscript?	In which section is the information available? (Reagents and Tools Table, Materials and Methods, Figures, Data Availability Section)

If study protocol has been **pre-registered, provide DOI in the manuscript**. For clinical trials, provide the trial registration number **OR** cite DOI.	Not Applicable	
Report the **clinical trial registration number** (at ClinicalTrials.gov or equivalent), where applicable.	Not Applicable	

Laboratory protocol	Information included in the manuscript?	In which section is the information available? (Reagents and Tools Table, Materials and Methods, Figures, Data Availability Section)
Provide DOI OR other citation details if **external detailed step-by-step protocols** are available.	Not Applicable	

Experimental study design and statistics	Information included in the manuscript?	In which section is the information available? (Reagents and Tools Table, Materials and Methods, Figures, Data Availability Section)
Include a statement about **sample size** estimate even if no statistical methods were used.	Yes	Quantification and statistical analysis (p.30)
Were any steps taken to minimize the effects of subjective bias when allocating animals/samples to treatment (e.g. **randomization procedure**)? If yes, have they been described?	Yes	Quantification and statistical analysis (p.30)
Include a statement about **blinding** even if no blinding was done.	Yes	Quantification and statistical analysis (p.30)
Describe **inclusion/exclusion criteria** if samples or animals were excluded from the analysis. Were the criteria pre-established?<br><br>If sample or data points were omitted from analysis, report if this was due to attrition or intentional exclusion and provide justification.	Not Applicable	
For every figure, are **statistical tests** justified as appropriate? Do the data meet the assumptions of the tests (e.g., normal distribution)? Describe any methods used to assess it. Is there an estimate of variation within each group of data? Is the variance similar between the groups that are being statistically compared?	Yes	Figure legends (p.22-25)

Sample definition and in-laboratory replication	Information included in the manuscript?	In which section is the information available? (Reagents and Tools Table, Materials and Methods, Figures, Data Availability Section)
In the figure legends: state number of times the experiment was **replicated** in laboratory.	Yes	Figure legends (p.22-25)
In the figure legends: define whether data describe **technical or biological replicates.**	Yes	Figure legends (p.22-25)

Ethics

Ethics	Information included in the manuscript?	In which section is the information available? (Reagents and Tools Table, Materials and Methods, Figures, Data Availability Section)
Studies involving **human participants**: State details of **authority granting ethics approval** (IRB or equivalent committee(s), provide reference number for approval.	Not Applicable	
Studies involving **human participants**: Include a statement confirming that **informed consent** was obtained from all subjects and that the experiments conformed to the principles set out in the WMA Declaration of Helsinki and the Department of Health and Human Services Belmont Report.	Not Applicable	
Studies involving **human participants:** For publication of **patient photos**, include a statement confirming that consent to publish was obtained.	Not Applicable	
Studies involving experimental **animals**: State details of **authority granting ethics approval** (IRB or equivalent committee(s), provide reference number for approval. Include a statement of compliance with ethical regulations.	Yes	Materials and methods (p.26)
Studies involving **specimen and field samples:** State if relevant **permits** obtained, provide details of authority approving study; if none were required, explain why.	Not Applicable	

Dual Use Research of Concern (DURC)	Information included in the manuscript?	In which section is the information available? (Reagents and Tools Table, Materials and Methods, Figures, Data Availability Section)
Could your study fall under dual use research restrictions? Please check biosecurity documents and list of **select agents and toxins** (CDC): https://www.selectagents.gov/sat/list.htm	Not Applicable	
If you used a select agent, is the security level of the lab appropriate and reported in the manuscript?	Not Applicable	
If a study is subject to dual use research of concern regulations, is the name of the **authority granting approval and reference number** for the regulatory approval provided in the manuscript?	Not Applicable	

Reporting

The MDAR framework recommends adoption of discipline-specific guidelines, established and endorsed through community initiatives. Journals have their own policy about requiring specific guidelines and recommendations to complement MDAR.

Adherence to community standards	Information included in the manuscript?	In which section is the information available? (Reagents and Tools Table, Materials and Methods, Figures, Data Availability Section)
State if relevant guidelines or checklists (e.g., **ICMJE, MIBBI, ARRIVE, PRISMA**) have been followed or provided.	Not Applicable	
For **tumor marker prognostic studies,** we recommend that you follow the **REMARK** reporting guidelines (see link list at top right). See author guidelines, under 'Reporting Guidelines'. Please confirm you have followed these guidelines.	Not Applicable	
For **phase II and III randomized controlled trials**, please refer to the **CONSORT** flow diagram (see link list at top right) and submit the CONSORT checklist (see link list at top right) with your submission. See author guidelines, under 'Reporting Guidelines'. Please confirm you have submitted this list.	Not Applicable	

Data Availability

Data availability	Information included in the manuscript?	In which section is the information available? (Reagents and Tools Table, Materials and Methods, Figures, Data Availability Section)
Have **primary datasets** been deposited according to the journal's guidelines (see 'Data Deposition' section) and the respective accession numbers provided in the Data Availability Section?	Yes	Data availability (p.31)
Were **human clinical and genomic datasets** deposited in a public access-controlled repository in accordance to ethical obligations to the patients and to the applicable consent agreement?	Not Applicable	
Are **computational models** that are central and integral to a study available without restrictions in a machine-readable form? Were the relevant accession numbers or links provided?	Not Applicable	
If publicly available data were reused, provide the respective **data citations in the reference list**.	Not Applicable	
